# Engineered CRISPR-Cas systems for the detection and control of antibiotic-resistant infections

**DOI:** 10.1186/s12951-021-01132-8

**Published:** 2021-12-04

**Authors:** Yuye Wu, Dheerendranath Battalapalli, Mohammed J. Hakeem, Venkatarao Selamneni, Pengfei Zhang, Mohamed S. Draz, Zhi Ruan

**Affiliations:** 1 Department of Clinical Laboratory, Sir Run Run Shaw Hospital, Zhejiang University School of Medicine, Hangzhou, China; 2 Department of Medicine, Case Western Reserve University School of Medicine, Cleveland, OH USA; 3 Department of Food Science and Human Nutrition, College of Food and Agriculture Sciences, King Saud University, Riyadh, Saudi Arabia; 4 Department of Central Laboratory, Shanghai Skin Disease Hospital, School of Medicine, Tongji University, Shanghai, China

**Keywords:** Bacteria, CRISPR-Cas, Antibiotic resistance, Therapy, Detection, Delivery

## Abstract

Antibiotic resistance is spreading rapidly around the world and seriously impeding efforts to control microbial infections. Although nucleic acid testing is widely deployed for the detection of antibiotic resistant bacteria, the current techniques—mainly based on polymerase chain reaction (PCR)—are time-consuming and laborious. There is an urgent need to develop new strategies to control bacterial infections and the spread of antimicrobial resistance (AMR). The CRISPR-Cas system is an adaptive immune system found in many prokaryotes that presents attractive opportunities to target and edit nucleic acids with high precision and reliability. Engineered CRISPR-Cas systems are reported to effectively kill bacteria or even revert bacterial resistance to antibiotics (resensitizing bacterial cells to antibiotics). Strategies for combating antimicrobial resistance using CRISPR (i.e., Cas9, Cas12, Cas13, and Cas14) can be of great significance in detecting bacteria and their resistance to antibiotics. This review discusses the structures, mechanisms, and detection methods of CRISPR-Cas systems and how these systems can be engineered for the rapid and reliable detection of bacteria using various approaches, with a particular focus on nanoparticles. In addition, we summarize the most recent advances in applying the CRISPR-Cas system for virulence modulation of bacterial infections and combating antimicrobial resistance.

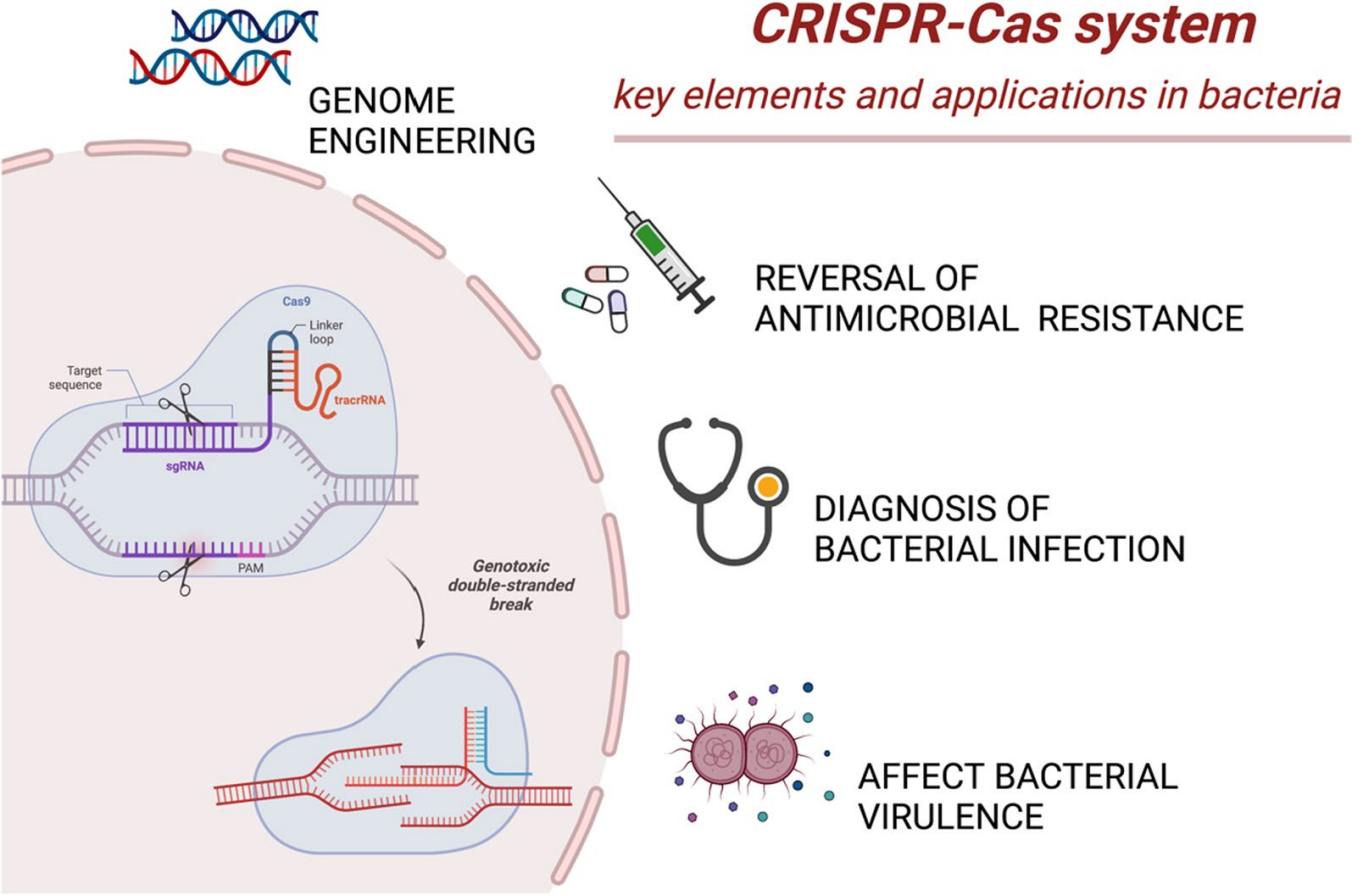

## Introduction

The discovery of antibiotics remains one of the most significant milestones in medicine and has widely enabled humans to prevent and treat bacterial infections. However, the misuse of antibiotics has fueled the emergence and spread of antibiotic resistance [1]. Multidrug-resistant bacteria are now one of the most alarming threats to global health, and they have attracted significant effort and resources worldwide [2, 3]. In 2017, the World Health Organization (WHO) defined a list of the most dangerous antibiotic-resistant bacteria that trigger community- and hospital-acquired infections titled ESKAPE (*E. faecium*, *S. aureus*, *K. pneumoniae*, *A. baumannii*, *P. aeruginosa*, and *Enterobacter* spp.) [4, 5], and these bacteria are called "superbugs" mainly due to the limited ability of antibiotics to treat their infections. These superbugs can share their genetic elements of resistance with other non-resistant bacterial species, accelerating the spread of antimicrobial resistance (AMR). It is necessary to discover and develop novel antibiotics and alternative treatment solutions to combat antibiotic-resistant bacteria [6]. Consequently, it is imperative to harness emerging techniques for the rapid diagnosis and treatment antibiotic-resistant infections using clinical specimens.

Accurate detection is always the first and key step in any strategy to control AMR and generally depends on detecting pathogen-specific markers, such as antibodies or nucleic acid sequences. Current detection protocols using polymerase chain reaction and immunoassays are time-consuming and require complex procedures and specialized instruments [7–9]. In addition, the detection of low concentrations of biomarkers using these methods remains challenging, especially in the early stage of infection, limiting our ability to slow and prevent the spread of AMR.

When foreign genetic elements (such as bacteriophages) invade bacteria, an immune response is built based on a defense mechanism in bacterial cells that uses the widely reported CRISPR (clustered regularly interspaced short palindromic repeats)-Cas system [10]. CRISPR and its associated genes (CRISPR associated, Cas) have the potential to provide a promising solution to combat antibiotic resistance [11]. A typical CRISPR-Cas system consists of three parts: i) a leading sequence, ii) an operon containing a group of Cas genes, and iii) a CRISPR DNA array. The CRISPR-Cas system has been rapidly deployed for genome editing in various cell types and experimental setups [12]. Observations from experimental trials have revealed its utility in targeting antibiotic resistance genes (ARGs) with high sensitivity and specificity rates. This illustrates the potential of the CRISPR-Cas system for use in developing novel antimicrobial agents and diagnostic tools for bacteria. The success rate of a bacterial genome editing technique partially depends on efficient delivery of the CRISPR system. Current advancements in nanotechnology have led to the development of nanoparticles with relatively large functional surface areas, biocompatibility, and unique optical and chemical properties that are suitable for efficient drug delivery. Therefore, nanoparticle-mediated molecule sensing and drug delivery were found to be a robust and promising approach for accurate pathogen diagnosis [13–15]. In addition, a non-viral delivery strategy for the CRISPR-Cas system would greatly promote its future therapeutic utility.

Previous studies show that significant effort has been made to engineer CRISPR as a rapid, accurate and smart tool for precise bacterial diagnosis [16, 17]. However, the integration of nanoparticles with the CRISPR system is still in its early stage, and wide research is required to enhance its application in treating viral and bacterial infections and restricting the spread of antimicrobial-resistant bacteria [18]. Here, we discuss the structures of different CRISPR-Cas systems, their mechanisms of action, and CRISPR array identification tools. Then, we report newly developed technologies based on integrated nanoparticles and the CRISPR-Cas system for the detection of bacteria and AMR and describe how CRISPR can ultimately be used to modulate bacterial virulence and combat AMR. Furthermore, we discuss the challenges and limitations of CRISPR technologies in the fight against AMR and the need to continue exploring their engineering and applications.

## Description of CRISPR-Cas systems

### Discovery of CRISPR-Cas systems

The CRISPR-Cas system was first discovered when researchers studied the nucleotide sequence of the *iap* gene in *E. coli* [19]. Jansen et al. [20] later discovered a new repetitive DNA family in prokaryotic cells composed of repeat sequences (ranging from 21 to 37 bp) separated by unique sequence spacers of similar lengths (ranging from 20 to 58 bp) and named as CRISPR. In 2005, a protospacer adjacent motif (PAM) that determines the specificity of CRISPR was found in the 3' end of the target DNA [21]. Sapranauskas et al. [22] reported that active CRISPR coding genes can be transferred across genera and interfere with the invasion of foreign nucleic acids. They proposed CRISPR to reduce the uptake and transmission of plasmid-encoded adverse genetic elements. In 2012, CRISPR technology was used for gene editing. Jinek et al. [23] studied Cas9 and its CRISPR-derived RNA (crRNA). The Cas protein has endonuclease activity and can cut target DNA alone under the guidance of mature crRNA. Subsequently, Jiang et al. [24] applied CRISPR gene-editing technology to the genomes of *S. pneumoniae* and *E. coli*. They observed that the combination of recombinant plasmids and oligonucleotide sequences leads to precise mutations in the genome, which plays an auxiliary role in gene engineering technology. Subsequent studies showed that CRISPR-Cas is present in most archaea and bacterial genomes and can potentially be applied for gene editing in different organisms [25].

### Classification of CRISPR-Cas systems

**Fig. 1:**
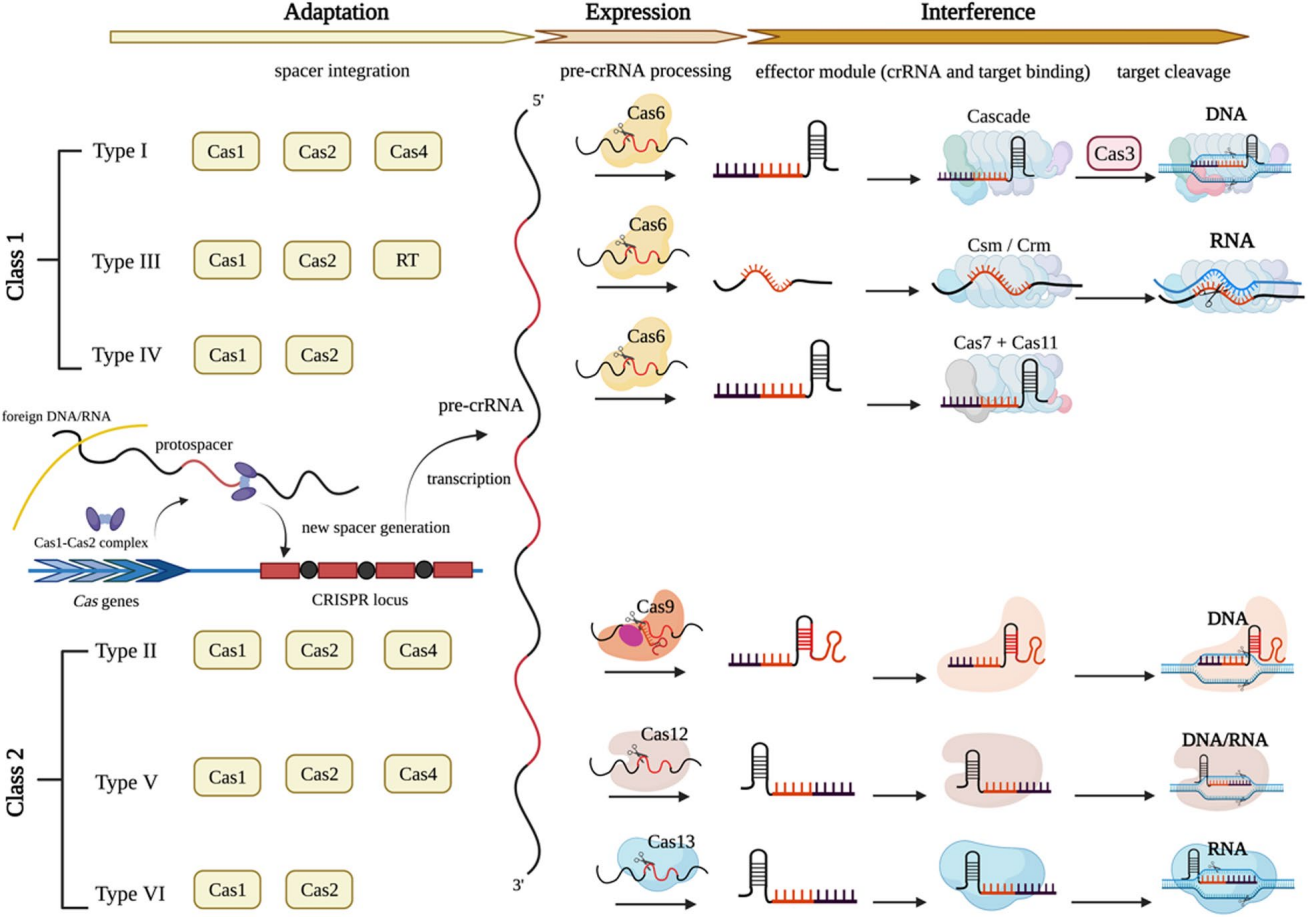
Classification and function of the CRISPR-Cas system in bacteria. Cas effectors are classified based on generic organization, and the functional modules of CRISPR-Cas systems are shown. (Adapted with permission from [26], and created with BioRender.com)

Accurate classification is essential for understanding the functions of these systems in bacteria and developing genome editing tools (Fig. 1). CRISPR-Cas systems can be divided into two classes, six types, and 33 subtypes based on the Cas protein composition and effect module sequence [25–27]. The effect module of Class 1 consists of multiple Cas proteins, including types I, III, and IV, with 16 subtypes—type I is split into seven subtypes (I-A to I-G); type III is divided into six subtypes (III-A to III-F); and type IV is divided into three subtypes (IV-A to IV-C). In comparison to the classification scheme presented in 2015, subtypes I-U were reclassified as I-G because the fusion of Cas4-Cas1 provides a new functional spacer in the I-U subtype system [28], and Cas3 occurs in all type I CRISPR-Cas systems and plays a role in eliminating the foreign target sequence. Type III contains Cas10 and can target DNA and RNA. Type IV contains Csf1, but it lacks the nuclease (Cas1 and Cas2) involved in targeted cleavage and includes no CRISPR sequence; thus, the system has no organization or function. However, multiple spacers targeting heterologous plasmids have been detected in type IV, implying that type IV can target plasmids and promote intracellular plasmid competition [29]. Class 2 consists of types II, V, and VI, with 17 subtypes. Among them, types II, V, and VI are composed of 3, 10, and 4 subtypes, respectively. Unlike Class 1, Class 2 is a single, large, multidomain crRNA binding protein that performs an interference function. Cas9 has the function of cutting DNA and processing crRNA, and it occurs in all type II systems. Both type V and VI CRISPR-Cas systems contain multiple variants, with type V Cas proteins mainly consisting of Cas12 (Cpf1) and Cas14 (now known as Cas12f), while type VI is Cas13a (C2c2) [26]. It is worth noting that type VI is a CRISPR-Cas system found to cut only targeted foreign RNA nucleic acid sequences [30].

### Mechanism of CRISPR-Cas systems in gene editing

The mechanism of the bacterial CRISPR-Cas system in protecting against invasion by foreign genetic material is divided into the following three steps: adaptation, expression, and interference [31] (Fig. 1). First, in type I and type II CRISPR-Cas systems, a 30-bp fragment of protospacer from the captured foreign DNA sequence is cut and inserted into the 5' end of the CRISPR-Cas site as a new spacer. To detect target DNA protospacers, it is necessary to select adjacent PAMs in advance [32, 33].

The second step involves crRNA expression. Different pre-crRNA maturation steps exist in a variety of CRISPR-Cas systems [34]. In type I, palindrome repeats in pre-crRNA must be transcribed to form a hairpin-like structure. With the help of Cas6 endonuclease, the 5' terminal spacer sequence of the hairpin-like sequence is separated, and a Cas6 protein maintains binding with each mature crRNA. In type II CRISPR-Cas systems, when foreign genetic material invades, pre-crRNA and tracrRNA (trans-activating RNA) are transcribed, and the latter provides a link between crRNA and Cas protein. A complex composed of pre-crRNA, tracrRNA, and Cas protein cleaves the RNA of the corresponding spacer to form crRNA [35]. In type III, the Cas6 complex cleaves the 3' terminal repeat sequence adjacent to the spacer of pre-crRNA, thus releasing the mature crRNA. In type V and VI CRISPR-Cas systems, Cas12 and Cas13 are involved in the expression of crRNA, respectively. However, the mechanism of crRNA maturation in type IV is still unclear.

crRNA and specific Cas proteins further combine to create a complex. In the third stage, i.e., secondary infection, the complex scans and identifies foreign genomes; subsequently, Cas nuclease cleaves the binding site to eliminate intruders. Type I involves assembling a complex of crRNA and Cas6 to eradicate invaders [35, 36]. In type II, the CRISPR-Cas system interferes with invaders by forming a complex of small guide RNA (sgRNA) and Cas9 to recognize the target DNA and cut it. Currently, the type II CRISPR-Cas system is extensively used in gene editing and diagnosis [37]. A PAM enhances the understanding of foreign genetic material in type I and type II systems [38]. In type III, crRNA binds with Csm (subtype III-A) and Cmr (subtype III-B) in the Cas protein, targets DNA and transcribes RNA cleaved by the Cas7 subunit, and then nonspecifically cleaves the remaining RNA by Csm6. In the type V CRISPR-Cas system, tracrRNA interferes with the Cas12b and Cas12c effector proteins (i.e., similar to Cas9) but not Cas12a. In type VI, the complex created by Cas13 and crRNA is targeted toward single-stranded RNA (ssRNA), and it does not require cooperation with tracrRNA and a PAM. However, binding of the Cas13 crRNA complex with complementary ssRNA requires the assistance of a protospacer flaking site (PFS), which is similar to a PAM on RNA [35].

### Detection of CRISPR-Cas in different genomes

With the rapid development of bioinformatics, an increasing number of scientists are turning their attention toward developing computational tools for the detection of CRISPR-Cas systems. The following specific CRISPR sequence identification tools, which mainly rely on repeating structures in arrays, have been created: CRT [39], CRISPRCasFinder [40], PILER-CR [41] and CRISPRDetect [42]. CRISPRDetect [42] is an extension strategy proposed by *k*-mer, and it annotates different types of sequence variation in nearly identical repeats. It determines which of the variant repeats in the spacer has a lower identity threshold, and it is more sensitive to short and degenerate repeats. However, CRISPRDetect has the disadvantage of incorrect segmentation when confronted with large overall CRISPRs. Mitrofanov et al. [43] developed CRISPRidentify, which is a CRISPR array detection method to process and evaluate repeat elements in the CRISPR-Cas system based on the machine learning method. Moreover, these researchers studied false-positive prediction problems in discovering CRISPR arrays. They proved that the process dramatically increases the sensitivity and specificity of CRISPR array recognition and decreases the false-positive rate.

With the increasing demand for CRISPR-Cas systems with different purposes, tools such as MetaCRAST [44], Crass [45], MinCED [46] and metaCRT [47] were deployed to explore the diversity and classification of CRISPR-Cas. Furthermore, a machine learning-based software tool called CRISPRCasTyper was developed to visualize gene maps for easy classification of diverse Cas operons and the occurrence of CRISPR-Cas subtypes in CRISPR arrays [48]. The tool was also implemented to perform crucial tasks such as identification and annotation of Cas loci based on recent nomenclature [26]. Additionally, other computational tools (i.e., CRISPRDetect, CRISPRleader and CRISPRSirection) have been specifically designed to predict CRISPR strands [42, 49, 50].

In addition to the aforementioned detection technologies, in silico methods are applied to design guide RNAs (gRNAs) with low off-target effects and high target effects. For instance, CRISPR-Local [51], GuideScan [52] and GPP sgRNA Designer [53] were developed based on CFD (computational fluid dynamics) technology [54] for quantifying off-target activity and are used to guide the design of sgRNAs. Additionally, crisprSQL [55] and OffScan [56] are used to detect off-target sites.

Therefore, a computer architecture pipeline integrating all of the abovementioned tools could become a cornerstone platform for researchers to automatically detect and classify CRISPR-Cas systems. Moreover, it would further provide assistance in understanding the full extent of CRISPR-Cas system applications.

## Use of CRISPR-Cas systems against pathogenic bacteria

### Direct killing of bacteria

**Fig. 2:**
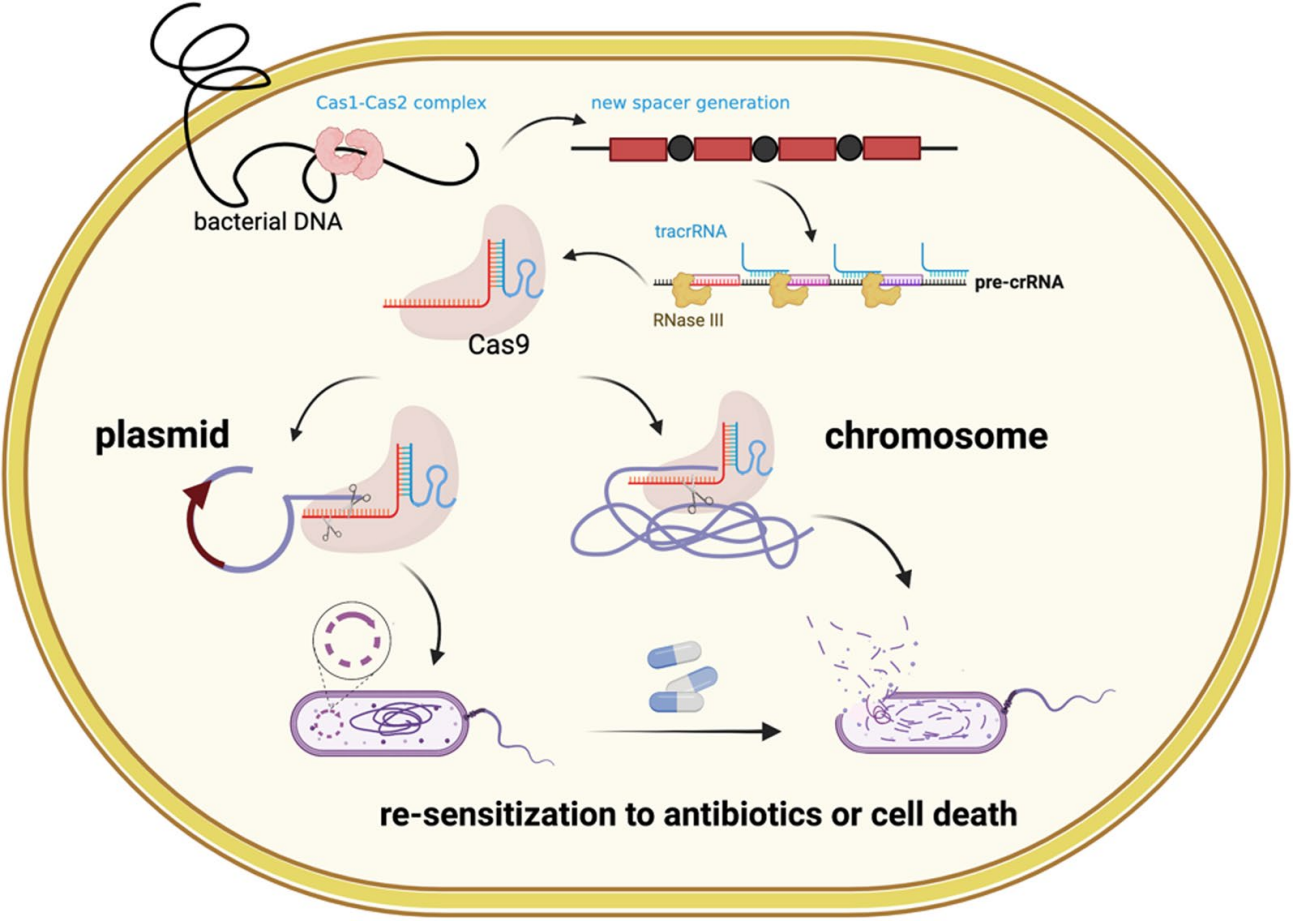
CRISPR-Cas systems as antimicrobials. The Cas9 RNA-guided nuclease is expressed together with guide RNA that will direct it to cut a target sequence. The target can be carried on a plasmid or/and the chromosome, leading to resensitization to antibiotics or cell death due to chromosome degradation. (Created with BioRender.com)

Unlike traditional antibiotics, which usually lack specificity, CRISPR-Cas directly and selectively attacks ARGs and eliminates pathogenic bacteria without affecting other bacterial species in complex bacterial populations [57] (Fig. 2).

The direct killing effect of the CRISPR-Cas system on pathogenic bacteria is due to its ability to target genes on chromosomes [58] and plasmids [59]. CRISPR-Cas9 plays a specific role in killing target genes on bacterial chromosomes, which has been confirmed in *S. aureus* [60, 61], *S. pneumoniae* [24, 62] and *Salmonella* [63]. Park et al. [64] selected the *nuc* gene unique to *S. aureus* as the target gene to verify the specific killing effect of the CRISPR-Cas9 system on the target bacteria. Further study indicated that the CRISPR-Cas9 bactericidal effect was mainly dependent on whether the system was effectively delivered to the target bacteria, and the researchers successfully eliminated *S. aureus* with high efficiency after delivering CRISPR-Cas9 via a phage carrier [64].

**Fig. 3:**
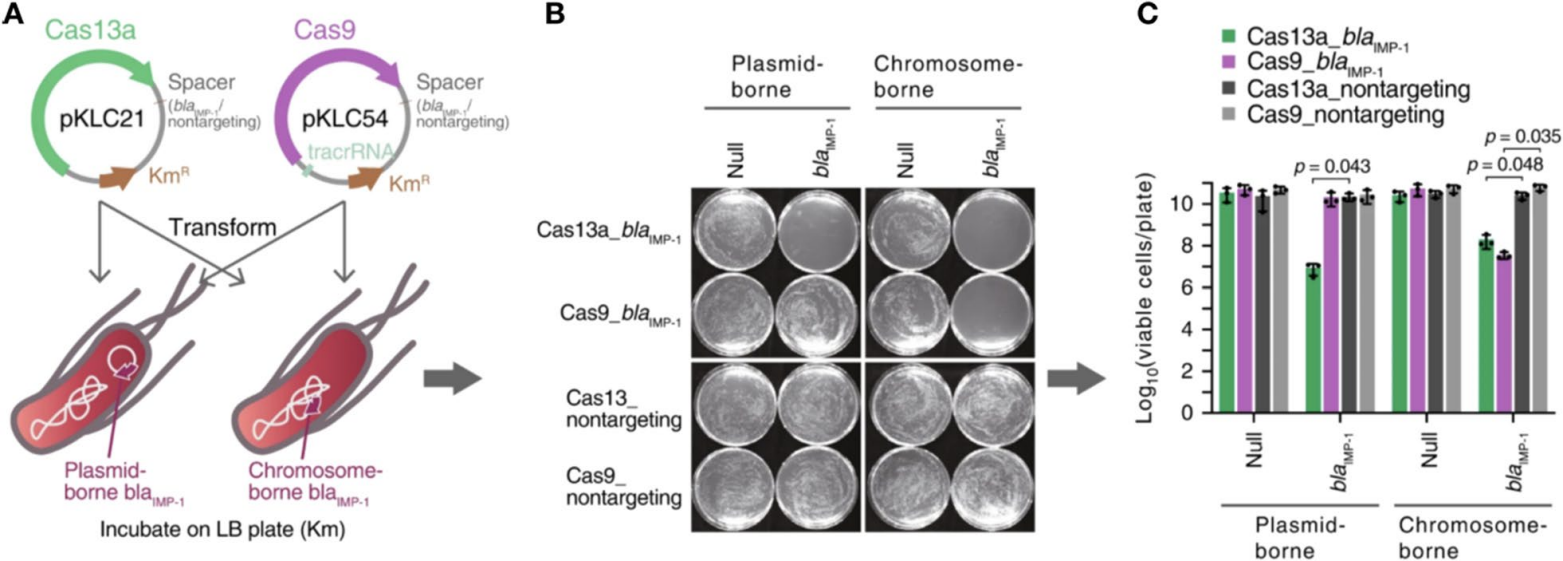
Sequence-specific bactericidal activity of CRISPR-Cas13a. **A** Schematic diagram of the transformation of CRISPR-Cas13a and CRISPR-Cas9 by targeting *bla*_IMP-1_ into *bla*_IMP-1_-expressing *E. coli* STBL3. **B** *E. coli* STBL3 expressing *bla*_IMP-1_ from a plasmid and chromosome was prepared and transformed with CRISPR-Cas13a or CRISPR-Cas9, both with a spacer targeting *bla*_IMP-1_ or no spacer (nontargeting). The resulting transformants were plated on an LB plate to test sequence-specific bacterial killing by CRISPR-Cas13a and CRISPR-Cas9. **C.** The number of bacteria on the plate obtained in experiment **B** was counted. Reprinted with permission from [65]

Gomaa et al. [63] reported that the type I-E CRISPR-Cas system could target the genome of *E. coli*. They used strains of *E. coli* with a high degree of homology in their genomes, *E. coli* K-12 and *E. coli* B, in which antimicrobial agents are often eliminated at the same time under specific conditions. They designed CRISPR spacers targeting the *fucP* gene and *ogr* gene, which exist in *E. coli* K-12 and *E. coli* B, respectively, to verify that the CRISPR-Cas system can achieve the targeted elimination of pathogenic bacteria while retaining symbiotic bacteria. Similarly, in 2020, the targeted killing effect of CRISPR-Cas against *E. coli* was also demonstrated by Kiga et al. [65]. The difference was that the sequence-specific bacteria-killing drug was based on CRISPR-Cas13a. Moreover, the bactericide based on Cas9 is only used to eradicate bacteria with the target gene on their chromosome. In contrast, the agent based on Cas13a kills bacteria with the target gene on both their chromosome and a plasmid. In the study by Kiga et al., the CRISPR-Cas13a system achieved a better bactericidal rate than CRISPR-Cas9 using an *E. coli* model with the carbapenem-resistant gene *bla*_IMP-1_ on both chromosomes and plasmids (Fig. 3). In addition, the CRISPR-Cas13a system does not directly cleave bacterial DNA. Nevertheless, it targets bacterial mRNA, which has lower mutation activity, indicating that the development and utilization of CRISPR-Cas13a as an antibacterial agent has excellent potential [65].

Selle et al. [66] used the endogenous type I-B CRISPR-Cas system of *C. difficile* as an antibacterial agent. The CRISPR-Cas3 system targets bacterial chromosomal DNA and causes bacterial death with the delivery of phages expressing bacterial genome-targeted crRNAs, effectively preventing and treating *C. difficile* infection (CDI). Recombinant phage engineering has a higher killing effect than wild-type phages. Correspondingly, bacteriophage delivery carriers of the CRISPR-Cas system act as antimicrobial agents, as described in detail below.

### Eradication of antibiotic resistance in bacteria

The CRISPR-Cas system also acts as an antimicrobial agent by restoring bacterial sensitivity to antibiotics by neutralizing ARGs (Fig. 2). In recent years, scientists have used the CRISPR-Cas system to target genes on the plasmids of pathogenic bacteria, especially ARGs, to resensitize drug-resistant bacteria to antibiotics. Studies have confirmed the role of the CRISPR-Cas system in resensitizing *S. aureus* to kanamycin [60] and methicillin [67]. In 2015, Yosef et al. [68] showed that the CRISPR system could simultaneously remove multiple plasmids carrying drug resistance genes. Subsequently, Kim et al. [69] searched for conserved sequences in TEM- and SHV-type ESBLs as targets for the powerful CRISPR-Cas9 system, producing a broad-spectrum β-lactamase of *E. coli*. It has been confirmed that CRISPR-Cas9 can altogether remove resistance gene plasmids (50–70 copies/cell) [59].

Interestingly, Tagliaferri et al. [70] further explored whether the CRISPR-Cas9 system removes small high-copy plasmids (100–300 copies/cell). They reported that the system only removed high-copy plasmids from some bacterial colonies. These scientists reported that treated bacteria regained sensitivity to resistance to ampicillin, cefazolin, cefuroxime, ceftriaxone, and cefotaxime. Liu et al. [71] used the CRISPR-Cas9 system to target and destroy *bla*_NDM-1_-encoding plasmids, and they showed that the CRISPR-Cas9 system is efficient against resistance plasmids, resulting in more than 99.99% target plasmid clearance within 8 h and lasting for 32 h. In a mouse model of skin and intestinal infection, the system restores the sensitivity of bacteria to kanamycin and effectively eliminates drug-resistant *E. coli*. Moreover, unexpected results show that the system can prevent the formation of anti-kanamycin mutations to ensure the effectiveness of antimicrobial agents.

However, for clinically complex multidrug resistance (MDR) plasmids, using a single nonessential target in cleansing can significantly reduce the effect. Therefore, the efficiency of the CRISPR-Cas system can be improved by designing a CRISPR-Cas system to target essential genes on resistance plasmids or establishing multiple cleavage sites using one CRISPR array or more than one sgRNA [72–74]. Rodrigues et al. [75] used the CRISPR-Cas9 system to target the *tet*(M) and *erm*(B) genes conferring tetracycline and erythromycin resistance to *E. faecalis*, respectively, which successfully reduced the antibiotic resistance of *E. faecalis *in vitro and in vivo. In the future, it is advisable to use CRISPR-Cas as an antibacterial agent for the remodelling of intestinal flora. Hao et al. [72] developed the pCasCure system based on the CRISPR-Cas9 system, which precisely cut and clear carbapenase genes such as *bla*_NDM_, *bla*_KPC_, and *bla*_OXA-48_ in carbapenem-resistant Enterobacteriaceae (CRE) and targeted *rep*A, *rep*B and *par*A on the pKpQIL plasmid to effectively clear the prevalent plasmid carrying the carbapenase resistance gene and resensitize CRE to carbapenem antibiotics. The MIC value was reduced by more than eight times.

Lysostaphin (Lst) is a staphylococcal lyase with strong application prospects. However, under the mediation of wall teichoic acids (WTAs), *S. aureus* developed resistance to Lst in the rich growth medium tryptic soy broth (TSB) [76]. Wu and his team used CRISPR-dCas9 to induce downregulation of the expression of *tar*H, *tar*O, and *tar*G genes, blocking the action of WTAs, making *S. aureus* less resistant to Lst, and eliminating the bacteria in TSB within 24 h [77]. Wu et al. [78] used the CRISPR-Cas9 genome-editing capability to edit and remove three drug resistance genes, *sul2*, *bla*_OXA-55_-like, and *nmcR*-like, in *S. algae*, which are resistant to carbapenem antibiotics. *S. algae* were then sensitized to sulfonamides, ampicillin, and imipenem. In addition, the CRISPR-Cas9 system can inactivate the *tet*(A), *ramR*, and *mgrB* genes, which affects the sensitivity of *K. pneumoniae* to tigecycline and polymyxin, as reported by Sun et al. [79].

**Fig. 4:**
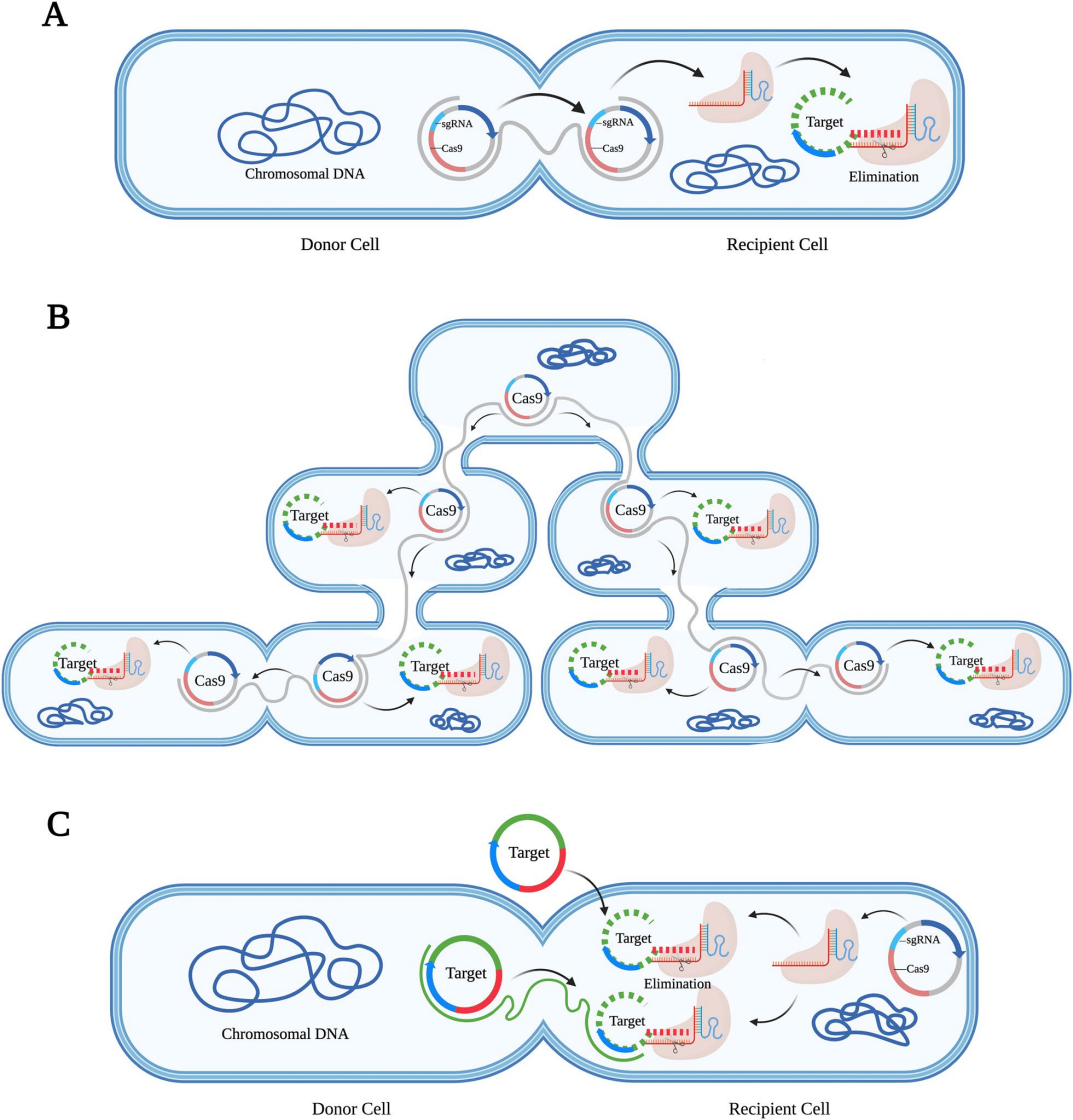
Schematic illustration of an engineered CRISPR-Cas9 system that targets antibiotic resistance genes. **A** The plasmid harbouring the *mcr-1* gene was destroyed by the conjugative CRISPR-Cas plasmid. **B** The engineered CRISPR-Cas9 was delivered to the target bacteria via a host-independent conjugative plasmid and continuously disseminated in microflora, affecting specific drug resistance plasmids containing the target sequence. **C** The engineered CRISPR-Cas9 system limits the conjugation and transformation of drug resistance plasmids in bacteria by targeting DNA. (Reproduced with permission from [81], and created with BioRender.com)

Plasmids that carry the drug resistance gene *mcr-1* cause resistance to polymyxin in MDR gram-negative bacteria [80]. Engineered CRISPR-Cas systems have great application potential in the clearance of this resistant plasmid. In the study by Dong et al. [81], for the first time, the engineered CRISPR-Cas9 system was combined with the host-independent plasmid vector pMob-Cas9 to target the *mcr-1* gene, avoiding the host-specific limitation in clearing the plasmid carrying the *mcr-1* gene and reversing the resistance of *E. coli* to polymyxin. Moreover, the plasmids could be transferred to the microbiome, persistently removing drug resistance plasmids that carry the *mcr-1* gene. In addition, the conjugated plasmid also prevented the horizontal transfer of the polymyxin-resistant plasmid pHNSHP45 and effectively prevented the transmission of the *mcr-1* gene [81] (Fig. 4). Similarly, Wan et al. [82] reported that the CRISPR-Cas9 system was able to reverse polymyxin resistance caused by the *mcr-1* gene in *E. coli* by building a high-copy number plasmid pUC19-*mcr-1* and recombinant plasmid pCas9-m1 or pCas9-m2 containing the sequence of sgRNA targeting the *mcr-1* gene to recognize the *mcr-1* gene and effectively remove plasmids harbouring *mcr-1*. *E. coli* were resensitized to polymyxin, and the elimination efficiency was greater than 80% at the 8th hour, lasting up to 24 h. The engineered CRISPR-Cas9 system also immunized *E. coli* against *mcr-1* [82]. Wang and his colleagues constructed the recombinant plasmid pMBLcas9-sgRNA, and the results showed that the genes involved in replication and distribution (*sop*A), binding (*nik*A), antibiotic resistance (*mcr-1*), and plasmid stability (*vag*C and *hic*B antitoxin genes) could be used as target genes for the clearance of drug resistance plasmids [73]. The study also showed that the engineered CRISPR-Cas system could eliminate plasmids in multiple strains step-by-step and eradicate multiple drug resistance plasmids simultaneously.

In addition to neutralizing resistance genes located on plasmids, the CRISPR-Cas system can also be applied to resistance genes on bacterial chromosomes. Using targeted gene modification mediated by the CRISPR-Cas9 system, Qiu and colleagues produced mutations in the *gyrA* gene that altered amino acids 83 and/or 87 of quinolone-sensitive *E. coli* ATCC strains, including one antibiotic-resistant strain, completely reversing the characteristics of quinolone resistance [83]. The causal relationship between *gyrA* mutation in *E. coli* and its resistance to quinolone antimicrobial agents was confirmed.

The CRISPR-Cas system effectively prevents the transformation of plasmids containing AMR genes, the transformation and conjugation of antibiotic-sensitive strains by plasmids, and the spread of drug resistance genes. Using methicillin-sensitive *S. aureus* as the research target, Bikard et al. [60] inoculated a phagemid to target the plasmid containing the tetracycline resistance gene and transferred the plasmid into the treated cells, but no tetracycline resistance was observed. Subsequently, Yosef et al. [68] delivered the CRISPR-Cas9 system into *E. coli* using λ phage as a vector and successfully destroyed the plasmids carrying the *bla*_NDM-1_ and *bla*_CTX-M-15_ genes. Moreover, compared with the negative control λ_cas,_ which lacks CRISPR, the plasmid transformation efficiency of these lysogens was greatly reduced to effectively prevent the transfer of antibiotic resistance elements [68]. In addition, Price et al. [84] performed research on multidrug-resistant *E. faecalis*, which usually lacks the CRISPR-Cas system, and they demonstrated that this kind of *E. faecalis* quickly obtains drug resistance genes due to the lack of genome defences. Rodrigues et al. [75] found that *E. faecalis* carrying CRISPR-Cas antibiotics can be immunized to resist the acquisition of antibiotic resistance. Therefore, CRISPR-Cas-based "vaccine" design to prevent drug resistance genes from entering antibiotic-sensitive bacteria is a method worthy of an in-depth study by scientists to prevent the spread of AMR via horizontal gene transfer (HGT). CRISPR-Cas system-mediated targeted elimination of antibiotic-resistant genes may become a potential tool for the clinical control of drug resistance gene transmission and drug-resistant pathogens.

### Delivery strategies

**Fig. 5:**
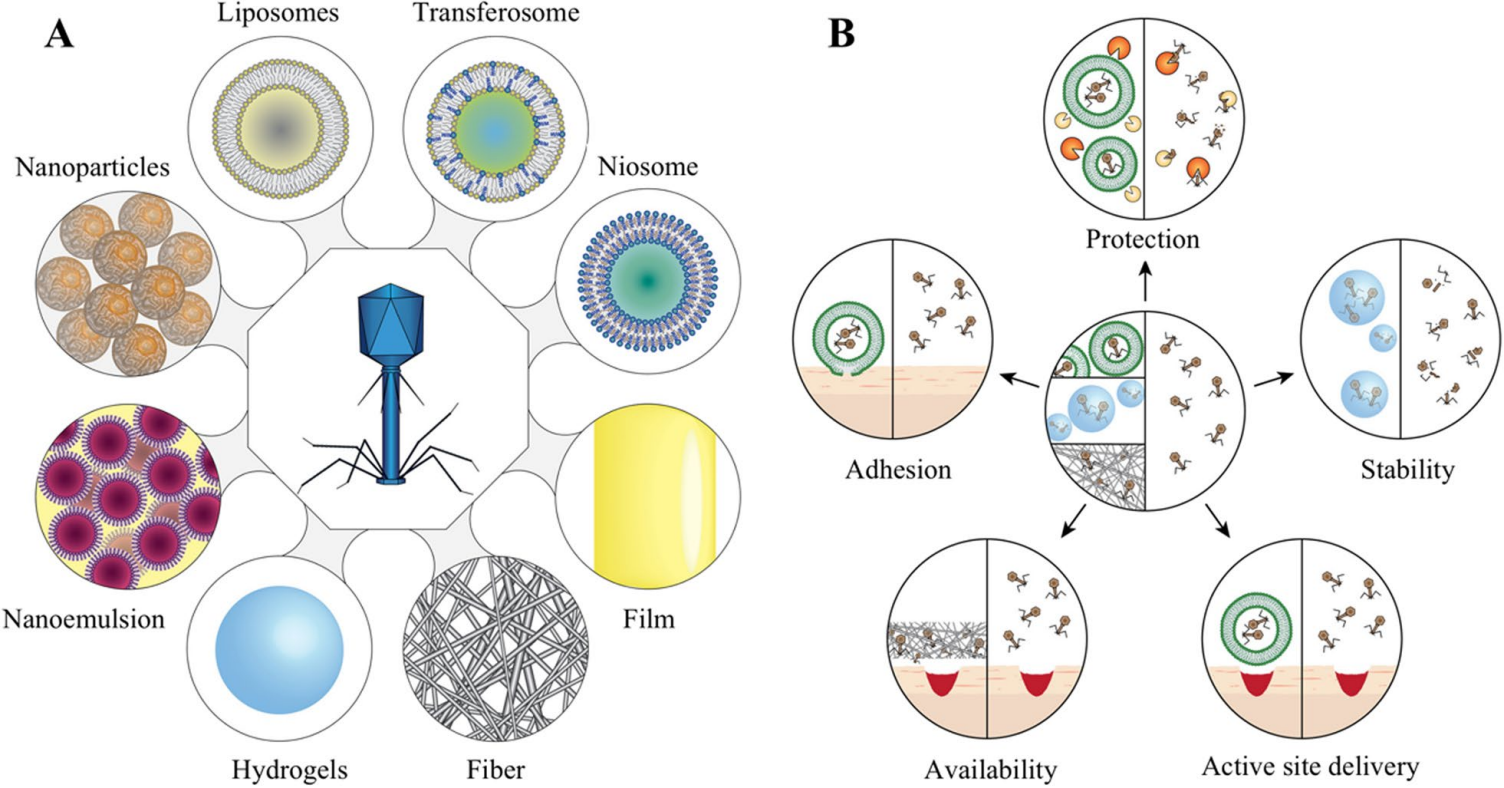
Methods and advantages of encapsulation by bacteriophages for therapeutic use. **A** Phage encapsulation methods. **B** Benefits of encapsulating phages for therapy versus the deployment of freely diffusing phages, including "protection" from conditions that inactivate the phage; "stability" during storage or administration of phages; "active site delivery" facilitation; and guaranteed "availability" to retain the phage at the site of infection and allow interaction with tissues to achieve "adhesion." (Reproduced with permission from [195])

How can an approximately 160 kDa protein-RNA complex be effectively delivered to the site of pathogenic bacteria to act as an antimicrobial agent? Several scientists have demonstrated the feasibility of using temperate phages to deliver the CRISPR-Cas system [59, 60, 64, 66, 68, 85]. They are designed to integrate the bacterial genes targeted by CRISPR into the genomes of temperate phages. A phage can inject its genome into bacteria to complete the invasion of bacteria. However, the phage-CRISPR-Cas system has a small host range, which has hindered its development. A study on *P. aeruginosa* has shown that single-nucleotide mutations in phage tail fibrin lead to host-specific changes [86]. Yosef et al. [87] used T7-derived defective phages to enhance DNA transduction to various bacteria by mutation of the tail fibre gene in the phage plasmid, allowing phage function loss to expand the host range. Perk and colleagues extended ϕSaBov host ranges by supplementing the gene encoding the tail fibre protein from ϕ11 (orf50) [64]. These results suggest that phage host specificity can be regulated by modifying the phage tail protein. Moreover, the bacteriophage-CRISPR-Cas system addresses only external and surface bacterial infections, increasing the complexity of some intracellular and tissue-specific bacterial infections. First, a bacteriophage-encoded CRISPR-Cas system would be able to selectively enter bacteria-infected cells. Second, phages must remain in the cell to function rather than being eliminated by the host's metabolic and immune pathways [88]. Therefore, phages can be encapsulated by chemical mediators (such as fibres [89, 90], liposomes [91–93], hydrogels [94, 95] and nanoemulsions [96]) to mediate the delivery of the phage-CRISPR system into the cell (Fig. 5). For example, liposomes are widely used as drug carriers, improving the stability, targeting, and long-term efficacy of encapsulated drugs, and they have the functions of presenting antigens and protecting antigens to prevent their degradation in vivo, with cationic liposomes exhibiting the best effect [97, 98]. Singla et al. [93] demonstrated that cationic liposomes, as bacteriophage delivery vectors, perform antibacterial functions as a promising drug delivery method. The results showed that this method protects the neutralizing antibodies of phages and removes bacterial biofilms. In addition, Cobb and colleagues delivered CRISPR-Cas9-modified phages using alginate hydrogel, demonstrating its ability to reduce soft tissue infection and increase its antibiofilm effect over time [95]. With the appropriate phage encapsulation strategy, the bacteriophage-CRISPR-Cas system can show highly effective antimicrobial activity.

**Fig. 6:**
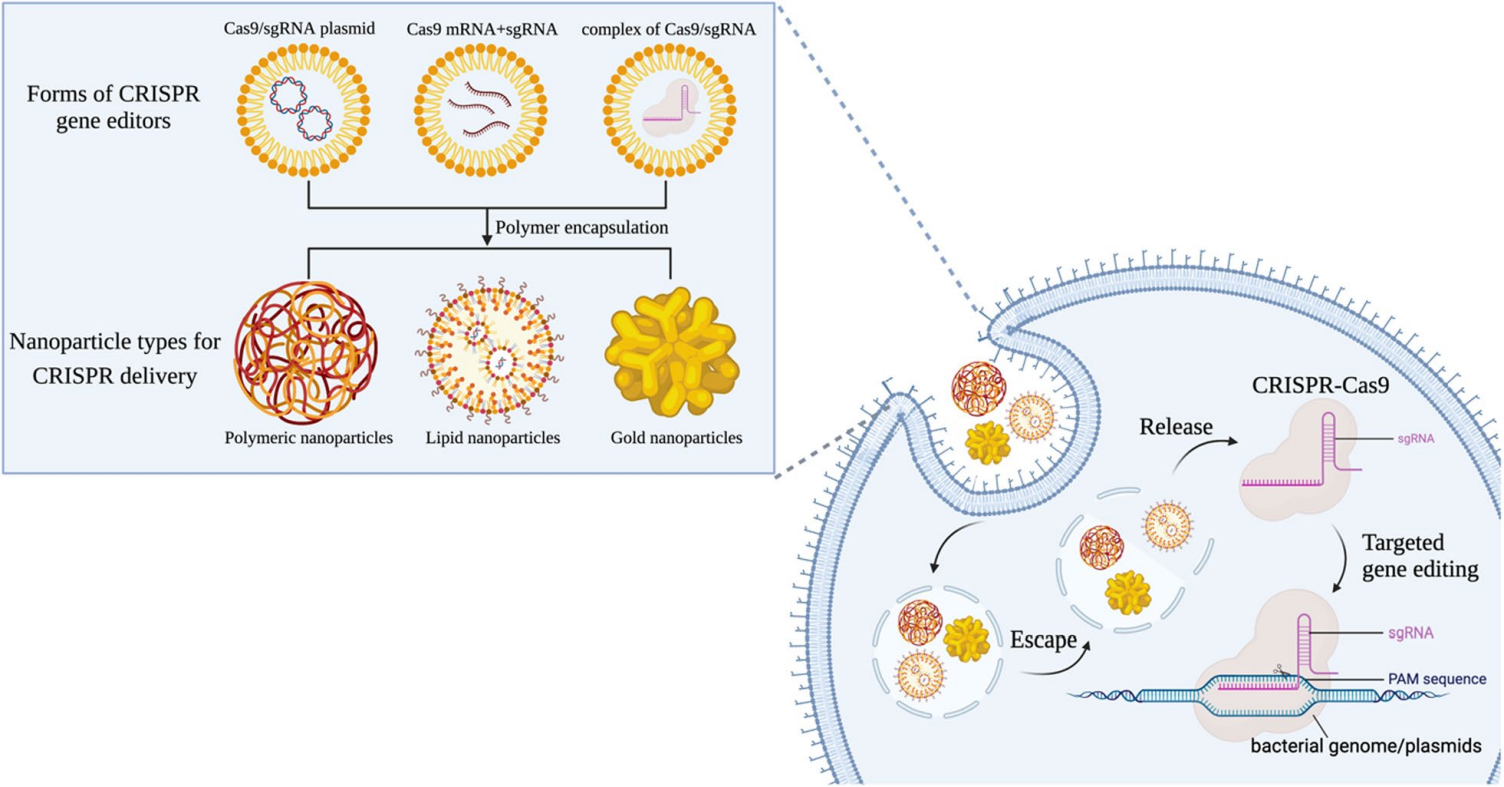
Nanoparticles for CRISPR-Cas9 delivery to combat bacterial infection. CRISPR gene editors are first encapsulated into nanoparticles in three forms: Cas9/sgRNA encoding plasmids, Cas9 mRNA and sgRNA, and complexes of Cas9/sgRNA. Three different types of nanoparticles are used to deliver CRISPR-Cas9, including polymeric nanoparticles, lipid nanoparticles and gold nanoparticles. Nanoparticles can help Cas9 reduce recognition and clearance by immune cells and effectively protect it from degradation. Then, the nanoparticles enter the target bacteria, release, and assemble into complete CRISPR-Cas9 systems. Finally, CRISPR-Cas9 performs gene editing in combination with the target gene sequence on the bacterial genome or plasmid to eliminate the bacteria. (Created with BioRender.com)

Although CRISPR-Cas systems offer promising results, there is still a long way to go to achieve clinical transformation of antimicrobial therapies based on CRISPR-Cas systems. Despite its advantages, this technique would overcome various obstacles to improve the efficiency of drug delivery [99]. Nanoparticles have been successfully used for drug and gene delivery [100–102], and engineering nanoparticle systems can be a practical and useful approach to deliver CRISPR-Cas9 (Fig. 6). Nanoparticle-based vectors have flexible sizes for packaging CRISPR-Cas systems and maintaining the natural state of their nanostructures during gene transfer. Additionally, they provide an anti-degradation barrier for nucleic acid molecules. At the same time, they also have the advantages of biocompatibility, surface functionalization, smaller immunogenicity and higher safety compared with virus vectors [103, 104]. Therefore, the non-viral vector gene delivery strategy has attracted wide attention in multiple areas [105].

Tao et al. [106] developed protamine-capped gold nanoclusters (AuNCs) as nanocarriers of CRISPR-Cas9, which efficiently deliver Cas9-sgRNA into target cells and accurately knock out the HPV E7 oncogene. The excellent fluorescence emission characteristics and adjustable surface functionalization of AuNCs also provide an imaging function of the nanoplatform to realize the role of real-time monitoring of biological effects. Suzuki et al. [107] developed a lipid nanoparticle-based CRISPR-Cas ribonucleoprotein delivery nanoplatform to inhibit HBV DNA and cccDNA in HBV-infected human hepatocytes. The platform avoided the loss of DNA cleavage activity, demonstrated excellent gene destruction and base replacement function, and did not exhibit any cytotoxicity. Kang et al. [67] developed nanosized CRISPR complexes (Cr-nanocomplexes). The recombinant Cas9 endonuclease from *Streptococcus pyogenes* (SpCas9) was directly covalently modified with branched polyethyleneimine (bPEI) to promote polymer insertion into bacteria and encapsulate sgRNA. Because of the high cationic property of bPEI and its possibility of causing the formation of molecular clusters, the efficiency of the complex into bacteria is enhanced. In addition, Cr-nanocomplexes use only a small number of carrier materials to minimize toxicity and side effects. The results showed that Cr-nanocomplexes targeting the *mecA* gene could effectively enter MRSA, and the complex had a high gene-editing ability to play an antibacterial role in drug-resistant bacteria.

Wu et al. [77] used electroporation technology to import a plasmid carrying CRISPR-dCas9 into *S. aureus* ATCC 6538 [108], which effectively restored the clearance effect of Lst on *S. aureus*. However, it is only suitable for in vitro tests, and the problems of cell damage and cytotoxicity remain. In addition, Ram and colleagues developed antibacterial community (ABD) systems that use engineered staphylococcal pathogenicity islands (SaPIs) to treat *S. aureus* infections. ABDs use SaPIs as the vector and insert CRISPR-Cas9, whose spacers target the *agr* gene. It has been demonstrated that ABDs directly kill infected *S. aureus*, and they have been tested in vivo. However, the stability of the system is problematic in the case of long-term operation [109].

**Fig. 7:**
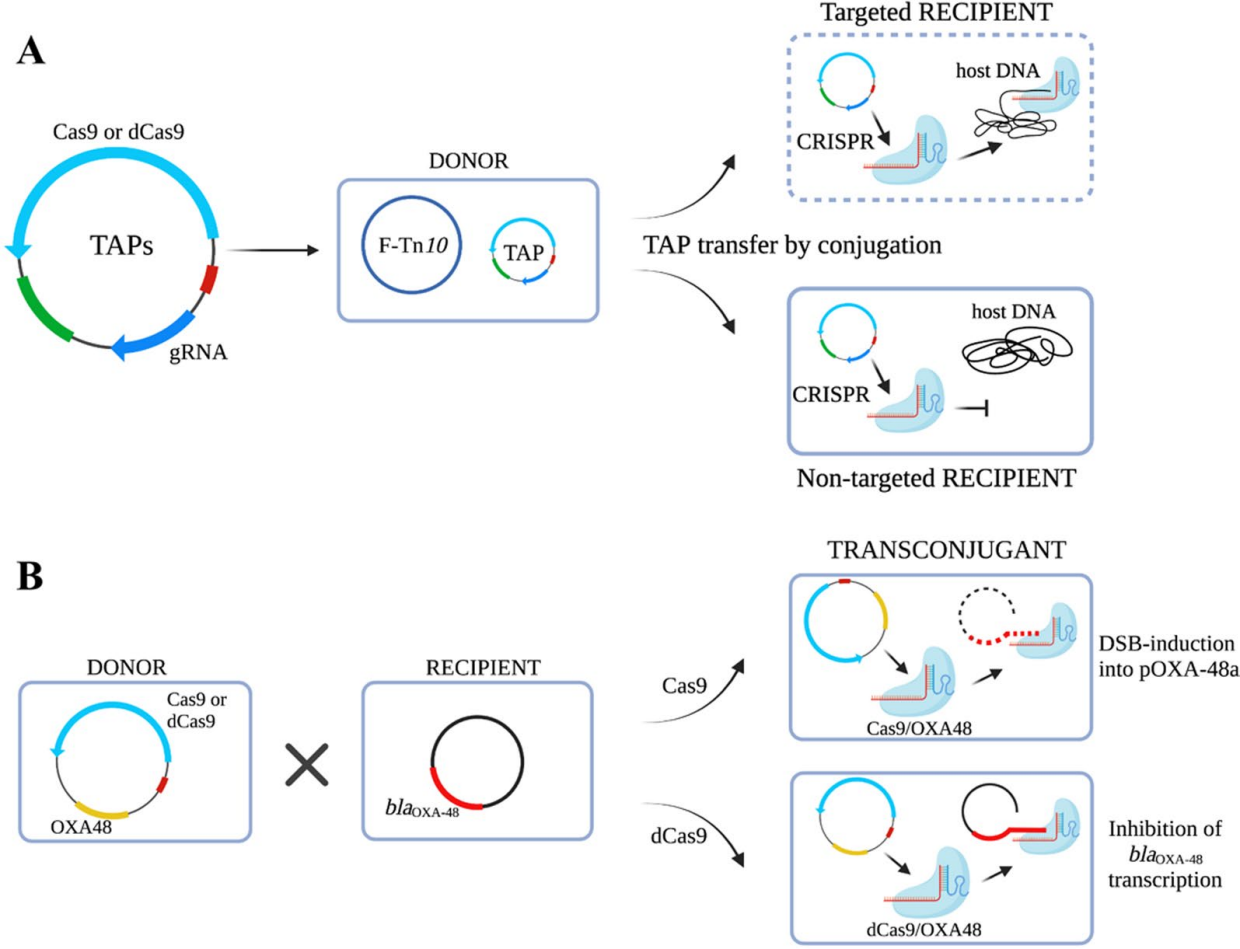
Schematic representation of targeted antibacterial plasmid (TAP) strategies. **A** The design of TAP modules and strategy of mediating killing of the target strain. **B** TAP resensitizes pOXA48-carrying recipient cells. (Reprinted with permission from [114], and created with BioRender.com)

Conjugative plasmids are an attractive strategy for CRISPR delivery, with the advantages of broad host ranges, no cellular receptors, and resistance to restrictive modification systems. Hamilton et al. [110] achieved the efficient interspecific conjugated transfer of CRISPR nuclease. Because the IncP RK2 system can bind to a wide range of different bacteria [111], these authors designed a system that delivers CRISPR nuclease in a complex microbial community. The high binding transfer frequency of the plasmid from *E. coli* to *Salmonella enteritidis* supports this view. Pheromone-responsive plasmids (PRPs) are plasmids that specifically propagate in and can fully penetrate the *E. faecalis* population. Their transmission is strictly regulated, thus making precise targeting and delivery possible [112, 113]. Rodrigues et al. [75] used PRPs to eliminate erythromycin-resistant *E. faecalis* in intestinal flora, demonstrating the ability of PRPs to precisely target resistant bacteria in intestinal flora, despite the low in vivo binding frequency. In 2021, targeted antibacterial plasmids (TAPs) were proposed (Fig. 7), which carry the CRISPR-Cas system and can be effectively transferred to *E. coli* and the highly related gram-negative Enterobacteriaceae. A TAP is only active against bacteria that contain DNA sequences complementary to the gRNA sequence that it carries, thus determining the ability of its specific target receptor strains to exert antimicrobial activity. These researchers also developed the CSTB algorithm, which is a bioinformatics method for reliably identifying gRNA to enable TAPs to target one or more target strains [114]. However, one drawback of the plasmid conjugation strategy is that it is less efficient. Scientists should focus on addressing this problem to better assist CRISPR-Cas in its antimicrobial action.

Table 1 shows various successful examples of successful CRISPR-Cas antimicrobial designs and the delivery strategies employed. From previous studies, it has been well conveyed that CRISPR-Cas systems have opened a new avenue to combat multidrug-resistant bacteria. Nevertheless, rigorous experimental work is needed to advance this technique for clinical trials. For instance, CRISPR-Cas systems need further testing to verify the efficacy toward wild-type clinical isolates. Attenuated strains could improve clinical safety during model establishment. Progress in nanotechnology may provide new solutions for the delivery of CRISPR-Cas systems to expand the safety and effective application of gene editing. Overall, a wide range of experimental trials is needed to annihilate CRISPR technology in combatting multidrug-resistant bacteria [72, 75, 115].

**Table 1:** Successful examples of designed CRISPR-Cas antimicrobials and delivery strategies

Delivery systems	Bacteria	CRISPR-Cas locus	Brief Result	Refs.
Phage	*E. coli*	λCas-CRISPR	Bacteria containing CRISPR-Cas targeting *bla*_CTX-M-15_ and *bla*_NDM-1_ genes had low transformation efficiency of AMR plasmids carrying these genes	[68]
	*S. aureus*	CRISPR-Cas9	A phagemid pDB91 targeting *mecA* was designed and encapsulated in phage ΦNM1, and MRSA was resensitized to methicillin	[60]
	*E. coli*	CRISPR-Cas9	RNA-guided nucleases induced double-strand breaks in *bla*_SHV-18_ or *bla*_NDM-1_, which reduced the transduction efficiency of plasmids containing these genes by approximately 1000-fold	[59]
	*E.coli*	CRISPR-Cas13a	CRISPR-Cas13a-induced bacteria decreased by approximately three orders and demonstrated sequence-specific killing activity against bacteria carrying the *bla*_IMP-1_ gene in an EC-CapsidCas13a_bla_IMP-1_ concentration-dependent manner	[85]
	*S. aureus*	CRISPR-Cas9	Integration of CRISPR-Cas9 system into ϕSaBov temperate phage genome, removal of *nuc* gene from the host chromosome, and expansion of host specificity of the phage was achieved by complementing the tail fibre protein	[64]
	*C. difficile*	CRISPR-Cas3, Type I-B CRISPR-Cas system	The endogenous type I-B CRISPR-Cas system in *C. difficile* redirects endogenous CRISPR-Cas3 activity against the bacterial chromosome. A recombinant bacteriophage expressing bacterial genome-targeting CRISPR RNAs was significantly more effective than its wild-type parent bacteriophage at killing *C. difficile*	[66]
Mobile genomic island	*S. aureus*	CRISPR-dCas9, CRISPR-Cas9	Highly mobile SAPIs were used to treat *S. aureus* infections, and ABD2003 killed *S. aureus* by introducing double-strand breaks in the *agrA* loci of the chromosome	[109]
Conjugative plasmid	*E. coli*	Types I-E CRISPR-Cas system	*E. coli* K-12 and B strains were removed by targeting *fucP* gene and *ogr* gene, respectively, and the two strains were removed by targeting the *groL* gene, demonstrating the sequence-specific removal function of CRISPR-Cas	[63]
	*E. coli, Salmonella*	CRISPR-Cas9	Plasmids based on the IncP RK2 conjugative system can be used as delivery vectors for a TevSpCas9 dual nuclease. Targeting of single or multiplexed sgRNAs to non-essential genes resulted in high *S. enterica* killing efficiencies	[110]
	*E. coli*	CRISPR-Cas9	An innovative strategy based on targeted-antibacterial-plasmids (TAPs) that uses bacterial conjugation to deliver CRISPR-Cas systems exerting a strain-specific antibacterial activity. TAPs directed against a plasmid-borne carbapenem resistance gene efficiently resensitized the strain to the drug	[114]
	*E. coli*	CRISPR-Cas9	The conjugative plasmid was used to deliver the CRISPR-Cas9 system targeting the *mcr-1* gene, restoring sensitivity to polymyxin in *E. coli*	[82]
Conjugative plasmid	*E. coli*	CRISPR-Cas9	The pMob-Cas9 plasmid carrying the CRISPR-Cas9 system was conjugated to *E. coli* for targeted clearance of the *mcr-1* gene	[81]
	*S. algae*	CRISPR-Cas9	CRISPR-Cas9 is used to target the *sul2*, *bla*_OXA-55_-like, and *NmcR*-like genes, making *S. algae* less resistant to carbapenem antibiotics	[78]
	*E. coli*	CRISPR-Cas9	*sopA*, *nikA*, *mcr-1*, *vagC*, and *hicB* antitoxin genes were used as target genes for the clearance of drug-resistant plasmids	[73]
	*E. faecalis*	CRISPR-Cas9	Description of the adaption of type II CRISPR-Cas system encoded on a pheromone-responsive conjugative plasmid that was efficiently transferred to *E. faecalis* for the selective removal of *ermB* and *tetM*	[75]
Hydrogel	*S. aureus*	CRISPR-Cas9	Quantitative antibiofilm effects increased over time for Fosfomycin-phage (dual) therapeutics delivered via alginate hydrogel. This module was successfully used to reduce soft tissue infection but not bone infection	[196]
Electroporation	*S. aureus*	CRISPR-dCas9	Electroporation technology was used to deliver CRISPR-dCas9 into *S. aureus*, inducing downregulation of *tar*H, *tar*O, and *tar*G genes and making the bacteria sensitive to lysostaphin	[77]
Nanoparticle	*S. aureus*	CRISPR-Cas9	The transfection efficiency of MRSA was significantly improved by mixing SpCas9-bPEI with sgRNA to form a nanosized CRISPR complexes (= Cr-Nanocomplex) designed to target *mecA*, which is a major gene associated with methicillin resistance	[67]

### Bacterial virulence

Virulence refers to the pathogenicity and infectivity of pathogenic bacteria. Recent studies have shown that bacterial pathogenicity is mainly controlled by phage invasion and binding plasmids. The CRISPR spacer regions targeting these mobile elements are related to the acquisition of virulence factors [116–118]. We studied the relationship of the CRISPR-Cas system with pathogen virulence to determine whether there was a positive or negative correlation (Table 2).

**Table 2:** Relationships between the CRISPR-Cas system and bacterial virulence

Function	Brief Result	CRISPR-Cas locus	Bacteria	Refs
Enhanced virulence	CRISPR-Cas system prevents bacteria from forming strong virulent strains with a capsule	CRISPR1 locus of *S. pyogenes*	*S. pneumoniae*	[62]
	Cas9 mediates the immune escape of TLR2, which increases the toxicity of bacteria	CRISPR-Cas9 system	*F. novicida*	[197]
	The deletion of CRISPR promotes the insertion of virulence genes and enhances virulence	CRISPRs of *V. parahaemolyticus*	*V. parahaemolyticus*	[127]
	There is significant correlation between the virulence factor *tdh* gene and the CRISPR-Cas system	Type II CRISPR-Cas system (subtype I-F)	*V. parahaemolyticus*	[126]
	Lack of CRISPR promotes the insertion of prophages from HGT	CRISPRs of *V. parahaemolyticus*	*V. parahaemolyticus*	[128]
	CRISPR systems resist phage invasion, regulate bacterial virulence and biofilm formation, and promote the evolution of *L. monocytogenes* towards high virulence	RliB-CRISPR, *CRISPR I-B and CRISPR II-A of L. monocytogenes*	*L. monocytogenes*	[198]
	RliB-CRISPR forms a stem-ring structure and regulates the virulence of bacteria	RliB-CRISPR	*L. monocytogenes*	[199]
	There is no correlation between the I-E CRISPR-Cas system and virulence genes, but the total number of spacer regions is negatively correlated with potential pathogenicity	CRISPR1- CRISPR4 (subtype I-E)	*E. coli*	[122]
	There is a negative correlation between the number of I-E CRISPR loci and pathogenic traits. Higher numbers of virulence factors result in lower repeat contents	CRISPR2 (subtype I-E)	*E. coli*	[123]
	The absence or presence of I-F system in bacteria may affect the distribution of virulence or ARGs	CRISPR-Cas system (subtype I-F)	*E. coli*	[125]
	The CRISPR system prevents the acquisition of some virulence factors, which is negatively correlated with the existence of some virulence factors	CRISPR1-*cas*, orphan CRISPR2, and CRISPR3-*cas*	*E. faecalis*	[200]
	Cas3 gene deletion mutant strains have increased virulence	Type I CRISPR-Cas3 system	*P. gingivali*	[131]
	Phage resistance may be related to low virulence, which makes non-phage-resistant strains more virulent	CRISPR-Cas system (*cas1, cas3-cas2,* and *cas6*)	*A.baumannii*	[130]
	The active CRISPR system of *B. thuringiensis* strains hinders HGT, including the transfer of virulence genes. Therefore, they have lower virulence than strains without an active CRISPR system	CRISPR-Cas system (subtypes I-C) of *B. cereus* strain	*B. cereus*	[129]
Reduced virulence	The expression level of several virulence genes in Cas3-deficient *S. mutants* is decreased	CRISPR1 system (type II-A) and CRISPR2 system (type I-C)	*S. mutans*	[201]
	The deletion of csn2 in *S. mutants* has multiple effects on pathogen virulence through gene expression changes	CRISPR-Cas9 system (csn2 gene)	*S. mutans*	[202]
	Inactivation of the *csn1* gene reduces the virulence of cst-II positive *C. jejuni* isolates	Type II CRISPR-Cas system	*C. jejuni*	[135]
	The virulence, adhesion ability, and survival ability of Δcas9 mutant strains are lower than those of wild-type strains	Type II CRISPR-Cas9 system	*C. jejuni*	[136]
	PA14 changes the virulence of bacteria by targeting and inhibiting LasR, and the bacteria has the ability to escape host defences	Types I-F CRISPR-Cas system of PA14	*P. aeruginosa*	[132]
	The presence of an active CRISPR-Cas system is associated with increased virulence	CRISPR-Cas systems (subtypes I-F, I-E, I-C)	*P. aeruginosa*	[203]
	*P. aeruginosa* maintains its CRISPR Cas system by inhibiting its toxicity	CRISPR-Cas system of PA14	*P. aeruginosa*	[204]
	The ΔCas9 mutant strains constructed with high-virulence clinical strains have low virulence, invasiveness, and adhesion ability	Type II CRISPR-Cas9 system	*Group B Streptococcus*	[205]
	Cas3 is involved in *Salmonella* biofilm formation and bacterial invasion, and it activates virulence	Type I CRISPR-Cas3 system (subtype I-E)	*Salmonella*	[133]
	Strains with the I-E* CRISPR-Cas system have higher virulence	CRISPR-Cas systems (I-E and I-E*)	*K. pneumoniae*	[206]

The CRISPR-Cas system has been demonstrated to interfere with the transformation and stability of plasmids with virulence genes [62, 119, 120]. The degradation of the CRISPR system is conducive to the recombination of a bacterial genome, thus enhancing its virulence [121]. The CRISPR sequences of 194 Shiga toxin–producing *E. coli* (STEC) strains with 43 serotypes were studied by Toro et al. [122], who discussed the potential relationship between the number of CRISPR and virulence genes. The results showed a significant negative correlation between the number of spacers in the CRISPR-Cas system and the pathogenic potential of STEC strains. Compared to strains with lower pathogenic potential, the number of CRISPR spacer regions in strains with higher pathogenic potential was lower. Similar results have also been reported by García-Gutiérrez et al. [123], Hong et al. [124] and Long et al. [125]. Thus, the absence of the CRISPR system in bacteria will affect *E. coli* virulence. *V. parahaemolyticus* is a gram-negative bacterium that causes acute gastroenteritis. Studies have shown that the *tdh* virulence factor of *V. parahaemolyticus* is significantly correlated with the existence of the CRISPR system in this strain [126]. Moreover, in the absence of CRISPR, phages enhance the virulence of *V. parahaemolyticus* by inserting virulence genes through horizontal transfer [127]. In addition, in studies of seven V*. parahaemolyticus* strains (five VP_AHPND_ strains and two non-VP_AHPND_ strains), some scientists have also found that CRISPR deletion promotes phage insertion [128]. Zheng et al. [129] found that almost all *B. cereus* groups do not contain or contain an incomplete CRISPR-Cas system. The existence of this system hinders HGT in bacteria. When this system does not exist, it is not only conducive to the acquisition of mobile genetic elements (MGEs) to improve the adaptability of this kind of bacteria to the environment but also advantageous for the acquisition of virulence factors by *B. thuringiensis* to improve its ability to infect the host. Leungtongkam et al. [130] demonstrated that phage host specificity is related to the geographic region because *A. baumannii* is more sensitive to phages from the same region. PCR detection of the Cas genes and virulence genes of *A. baumannii* has revealed that they are correlated. At the same time, phage-resistant strains are more toxic than nonphage-resistant strains [130]. The pathogenicity of *P. gingivalis* is related to the CRISPR-Cas protein Cas3, as discovered by Solbiati et al. [131] in 2020. Compared to the wild type, Cas3 mutants are more virulent. A *Galleria mellonella* infection model was established by infecting larvae with Cas3 mutants, and its virulence was significantly increased within 48 h.

In contrast, some reports have suggested a positive correlation between CRISPR's regulation of virulence and pathogenicity [132–134]. In 2013, Louwen et al. [135] showed that *cas2*, *cas1*, and *csn1* gene mutations in the CRISPR-Cas system are related to the presence of Cst-II sialtransferase in *C. jejuni* isolates, and they also inactivated the *csn1* gene, resulting in almost complete loss of the virulence gene of Cst-II-positive *C. jejuni* because Cas9 was incomplete. In a study of the pathogenesis of *C. jejuni*, Shabbir et al. [136] compared *C. jejuni* NCTC11168 with the ΔCas9 mutant and found that the presence of the Cas9 gene promotes biofilm formation, enhances virulence, regulates the adhesion and invasion genes of *C. jejuni* in host epithelial cells and promotes its ability to reproduce and survive in macrophages. To determine the virulence of *Salmonella*, scientists have compared Cas3 WT *Salmonella* strains to Cas3 mutant strains and found that the WT strain has stronger cell invasiveness and viability. In an established chicken infection model, the virulence of the Cas3 mutant strain was lower, and the strain carrying Cas3 had a higher mortality rate in chickens [133].

Based on the above findings, the CRISPR system is related to bacterial virulence; thus, analysis of the CRISPR sequence is helpful in studies of the mechanisms of bacterial virulence changes. The role of these systems in regulating virulence is undoubtedly a new and exciting research field. However, due to the limitation of the number of strains, isolation area, and time, there is no unified assessment of the relationship between bacterial CRISPR and virulence, indicating that bacterial CRISPR and virulence should be further studied. Understanding the mechanism of virulence control exerted through the CRISPR-Cas system will provide a deeper perspective on gene regulation. Future studies should explain how these systems promote the pathogenesis of bacteria, which will help identify bacteria and provide defence strategies during infection.

## Application of CRISPR-Cas for the detection of bacterial infections

**Fig. 8:**
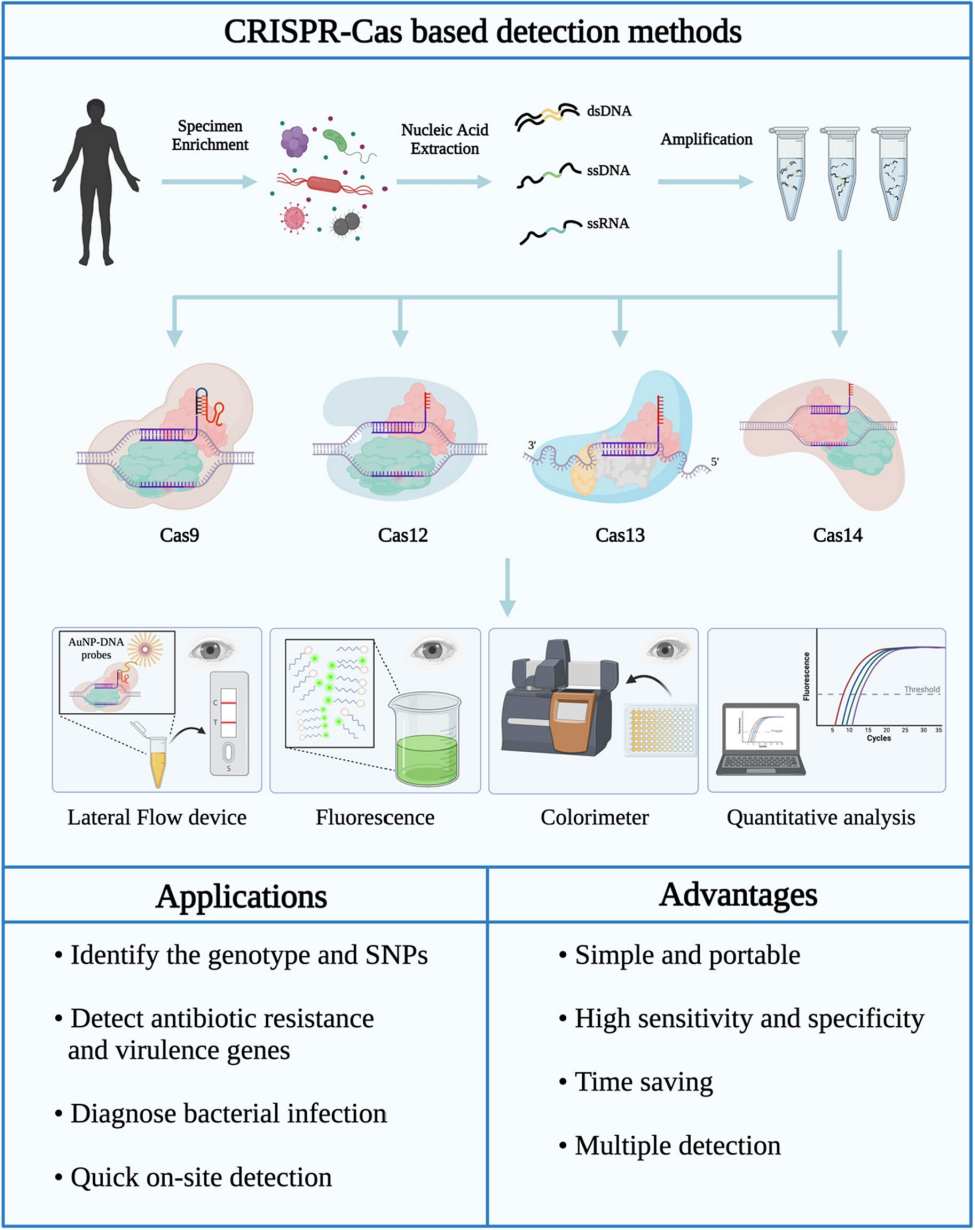
Steps, applications, and advantages of bacterial detection methods based on the CRISPR-Cas system. CRISPR-Cas systems (i.e., Cas9, Cas12, Cas13 and Cas14) have been developed for pathogen diagnosis. First, clinical samples are enriched, and pathogen nucleic acids are extracted and amplified. For RNA samples, reverse-transcription amplification with T7 transcription is required. Then, the target is identified and cleaved by the corresponding CRISPR-Cas system. The sensitivity can be enhanced by fluorescence or lateral flow assays, the results can be observed with the naked eye, and the quantitative detection of pathogens can be realized. In addition, CRISPR-Cas systems can be used for diverse diagnostic purposes, such as distinguishing pathogen genotypes from SNPs, distinguishing ARGs from virulence genes, diagnosing pathogen infections, and performing rapid on-site detection. CRISPR-based pathogen diagnostics facilitate simple portability, high sensitivity and specificity, time savings, and multiple detection. (Created with BioRender.com)

Several CRISPR-Cas systems have been developed to detect nucleic acids and biomarkers in bacteria and viruses [16]. They can be used to accurately identify genotypes and single nucleotide polymorphisms (SNPs) in pathogenic bacteria, detect ARGs and virulence genes in pathogenic bacteria, and diagnose bacterial infections [137]. With scientific progress in nanotechnology, a nanosized CRISPR complex can be used for quick on-site diagnosis [138, 139]. Compared to traditional nucleic acid detection technology, the CRISPR-Cas system has the following advantages. i) It is simple and portable and uses lateral flow assays that do not rely on special instruments or a specific environment, thus allowing on-site detection [140, 141]. ii) It is a time-saving technology that combines CRISPR-based reaction systems with fluorophores, quenchants and nanoparticles or turbidity changes, allowing results to be observed by the naked eye in only a few hours [142–144]. iii) This technique yields a better sensitivity and specificity rate than quantitative polymerase chain reaction (qPCR), which is often regarded as the gold standard [145]. iv) Additionally, it allows simultaneous detection of different target molecules [146] (Fig. 8). Therefore, the technology is now recognized as a novel approach for next-generation diagnostics that simultaneously meets multiple test criteria [16, 147].

### DNA-targeting CRISPR-Cas systems in diagnostics

Cas9 recognizes double-stranded DNA (dsDNA) and specifically cleaves complementary strands of DNA strands interacting with sgRNA through nucleic acid base pairing [148]. A technique called FLASH [147] uses CRISPR-Cas9 to implement the multiplexed detection of AMR sequences, which is used to test the resistance of *S. aureus* strains and is important in the detection of MRSA infection vancomycin-resistant *E. faecalis*. Müller et al. developed a single-step optical DNA mapping assay based on the fluorescent dye YOYO-1 and the AT-selective molecule netropsin. This method identifies the spread of a complete plasmid in a nosocomial outbreak regardless of whether the plasmid has a DNA sequence [149, 150]. Müller et al. [151] continued their study and successfully combined the single-step optical DNA mapping assay with CRISPR-Cas9 to directly identify specific resistance genes (*bla*_CTX-M_, *bla*_NDM_, and *bla*_KPC_) on plasmid molecules. Their test yields sequence information within hours, making it ideal for rapidly tracking infections. Subsequently, they used this technology to analyse the type of plasmid *bla*_CTX-M_ gene present during a nosocomial outbreak and discover the possibility of the presence of plasmid in specimens. Moreover, they reported no plasmid transfer in ESBL-producing *E. coli* in this nosocomial infection outbreak [145]. Recently, these scientists also developed novel DNA labelling techniques based on the CRISPR-Cas9 system. They demonstrated that designing a set of sgRNAs to enable CRISPR-Cas9 to specifically target any 20 bp sequence dramatically improves the detection of markers with specific characteristics [152]. Repeated sequences and other sequences that are difficult to access by markup methods can also be applied. In addition, their multiple sgRNAs provide more sequence information, which improves the accuracy of precise localization of structural variations. Using *H. influenzae* as a model, they used the CRISPR-Cas9-based fluorescent labelling technique to accurately identify single alleles affecting the 3'-NGG PAM site and even pinpoint single-base differences in highly conserved sequences with sequence motif-based labelling.

A strategy called CAS-EXPAR has been reported [153], which is based on the isothermal exponential nucleic acid amplification reaction mediated by CRISPR-Cas9 and nicking endonuclease (NEase). It has the advantages of rapid and site-specific nucleic acid detection, and it does not require exogenous priming, effectively avoiding the independent nucleic acid amplification caused by foreign primers. Furthermore, it can also be used to detect DNA methylation and *L. monocytogenes* total RNA [153]. In addition, the CRISPR-Cas9 system was also successfully applied in a study targeting the quinolone drug resistance gene *gyrA* mutation, which confirmed that the mutation of nucleotide 248 and 259 of *gyrA* gene caused the mutation of amino acids 83 and 87, thus leading to quinolone resistance in *E. coli* [83]. Therefore, the development of the CRISPR-Cas system as a research tool for the mechanisms of bacterial drug resistance has great prospects. Sun et al. [154] designed a fluorescence-sensing method based on CRISPR-Cas9 for bacterial detection, in which Cas9 cleaves target DNA to produce short ssDNA, followed by binding a round probe to its 3' terminus. Then, a long ssDNA copy of the circular probe is synthesized with the assistance of DNA polymerase, and quantitative detection of *E. coli* is performed with the assistance of the UIO66 platform based on fluorescence intensity. The platform has a high sensitivity and detection range, with a detection limit three orders of magnitude lower than qPCR method.

Cas12 has been used to exert nonspecific lytic activity against ssDNA by recognizing dsDNA [155]. Based on Cas12, the development of DetectR [155] has been promoted, which is highly sensitive, quickly detects individual DNA or RNA, and allows the identification of individual base mismatches. The "collateral cleavage" activity of CRISPR-Cas12 has also been used to diagnose *M. tuberculosis* in clinical samples [156] and to identify strains and subspecies of the bacterium [157]. In 2020, scientists demonstrated the diagnostic performance and early diagnostic value of CRISPR-Cas12 in paediatric tuberculosis [158]. In addition, Bonini et al. [159] developed the first portable biosensing device based on CRISPR-Cas12a with a collective label-free impedance assay for quick detection of *E. coli* and *S. aureus* with high sensitivity and specificity. The detection limit for *S. aureus* was as low as 3 nM. In addition, Curti et al. [160] applied CRISPR-Cas12a (known as Cpf1) to identify target sequences corresponding to carbapenem resistance genes, such as *bla*_KPC_, *bla*_NDM_, and *bla*_OXA_. Overall, with the label-free impedance assay, the detection time was reduced to less than an hour, and the results were comparable to those of qPCR (i.e., in terms of accuracy and sensitivity). In addition, the results are validated by portable test strips, which are cost effective and have a 100% correlation with results in fluorescence tests [160].

CRISPR-Cas14 is the smallest known Cas protein. It is an RNA-guided endonuclease that can target and cleave ssDNA [161]. Song et al. [162] combined it with the magnetic DNA nanoparticle system to establish a fluorescent nucleic acid detection platform that can perform diagnostic analysis without complex instruments and nucleic acid purification, which is known as the Cas-TSPE system. Cas14a was conjugated to target-specific primer extension (TSPE). Using only a general sgRNA to identify marker sequence-specific primers, the fluorescent DNA sensing platform can detect various pathogens, including *E. coli*, *S.* Typhi, *P. aeruginosa*, *S. aureus*, *S. pyogenes*, and *E. faecalis*.

Ge et al. [163] also used the function of Cas14a to establish a Cas14a1-mediated nucleic acid detection platform (CMP) for the rapid detection of pathogens in milk samples. This promoted the development of pathogen detection in the field of food safety. The Cas14a1-sgRNA complex binds and cleaves the target ssDNA, activating the collateral cleavage of the nonspecific ssDNA in the presence of fluorescent and quenching agents. In addition, they used CMP and qPCR simultaneously for the quantitative detection of *S. pyogenes* and *S.* Typhi in samples, and the results showed that CMP provided higher sensitivity than qPCR [163].

### RNA-targeting CRISPR-Cas systems in diagnostics

CRISPR-Cas13 recognizes and cuts ssRNA, indicating the presence of target RNA by releasing signals [30]. Taking advantage of this effect of Cas13, Kellner et al. [164] at the Broad Institute in 2017 developed a new technique called SHERLOCK for the detection of nucleic acids. It is a highly sensitive technique that detects viral particles at concentrations as low as 2 aM (2 × 10^–18^ mol/L). In the following year, they further improved the SHERLOCK technique and introduced SHERLOCKv2 [164], which is a system that simultaneously detects multiple viral nucleic acids in 90 min and is also suitable for identifying pathogens that cause pneumonia [165]. In the same year, the development of heating unextracted diagnostic samples to obliterate nucleases (HUDSON) enabled SHERLOCK to detect pathogens in patient samples at concentrations as low as 1 copy/μL while achieving similar speeds and equipment requirements to rapid antigen detection [166]. In 2020, a test system called APC-Cas [167] was proposed to sensitively, quantitatively, and selectively detect *Salmonella* in milk samples. In an in vivo mouse model, the APC-Cas system can distinguish between early- and late-stage *Salmonella*-infected mice and normal mice, illustrating the potential of the system for the early diagnosis of pathogenic bacteria. In 2021, the *lcrV* gene was found to be highly expressed in the early stages of *Y. pestis* infection and is considered a marker of pathogen infection [168]. However, the *lcrV* gene exists in a plasmid with a corresponding low copy number and cannot be recognized by qPCR. In 2021, Schultzhaus et al. [169] used collateral cleavage activity of the CRISPR-Cas13a system to realize the detection of the *lcrV* virulence gene. They proposed that when crRNA is carefully purified and added to the reaction system at a specific concentration during in vitro transcriptional reactions, they system performs better due to avoiding the possibility of inhibiting the performance of Cas13a and reducing the time required to screen crRNA sequences.

### Nanoparticles and CRISPR-Cas system-based diagnostics

The design, development, and application of novel functional nanomaterials has become a highly popular research field in biosensing, medical imaging, drug delivery, etc. [170–173]. In addition to the great potential of nanoparticles to participate in the targeted delivery of CRISPR-Cas systems as antimicrobial agents, they also have significant application value in the design and development of biological diagnostic tools based on CRISPR-Cas systems. In recent years, research on nucleic acid probes based on nanoparticles has become a focus, facilitating substantial progress in the diagnosis of viruses [174–176], cancers [177, 178], and pathogens [179, 180].

Nanoparticle-based probes show great potential for target detection due to their unique colorimetry, high fluorescence yield, and good light stability. Spherical nucleic acids (SNAs) based on AuNPs can significantly improve the stability of nucleic acid reporter molecules in the biological environment due to the negative charge and localized high concentration of salt ions on the surface of AuNPs. Additionally, AuNPs show high quenching efficiency [181, 182].

Hu et al. [183] achieved, for the first time, the successful construction of DNA/RNA-AuNP and DNA-HRP-AuNP probes using a single-step, salt-aging-free, and strong Authiol-free freezing-based labeling method within a few minutes. They developed Magnetic Pull-Down-Assisted Colorimetric Diagnosis Based on the CRISPR-Cas12a System (M-CDC) and successfully detected the *nuc* gene in *S. aureus*. The results showed that M-CDC has good specificity and detection limits. Subsequently, they designed a DNA-HRP-AuNP probe based on frozen markers that enhanced the M-CDC assay with HRP. Furthermore, the researchers added a reverse-transcription step in the experiment. They reverse transcribed the ssRNA of the virus into cDNA and successfully used Cas12a to achieve indirect detection of RNA [183]. Kim et al. [184] studied CRISPR-mediated surface-enhanced Raman spectroscopy (SERS) analysis, which combines CRISPR-Cas9 with SERS-active Au-coated magnetic nanoparticles (Au MNPs), and applied it to detect antimicrobial resistance genes. Based on the inherent SERS spectrum of bacteria and the high sensitivity of SERS, multiple and accurate identifications of bacteria can be realized [185, 186]. Au MNPs have the advantage of promoting the separation and concentration of targets [187]. This method successfully detected three MDR bacteria, *S. aureus*, *A. baumannii*, and *K. pneumoniae*, and was verified in a mouse infection model. In addition, these scientists applied a 3D nanopillar array swab, thereby omitting unnecessary sample preparation steps to realize on-site identification of MDR bacteria [184].

**Fig. 9:**
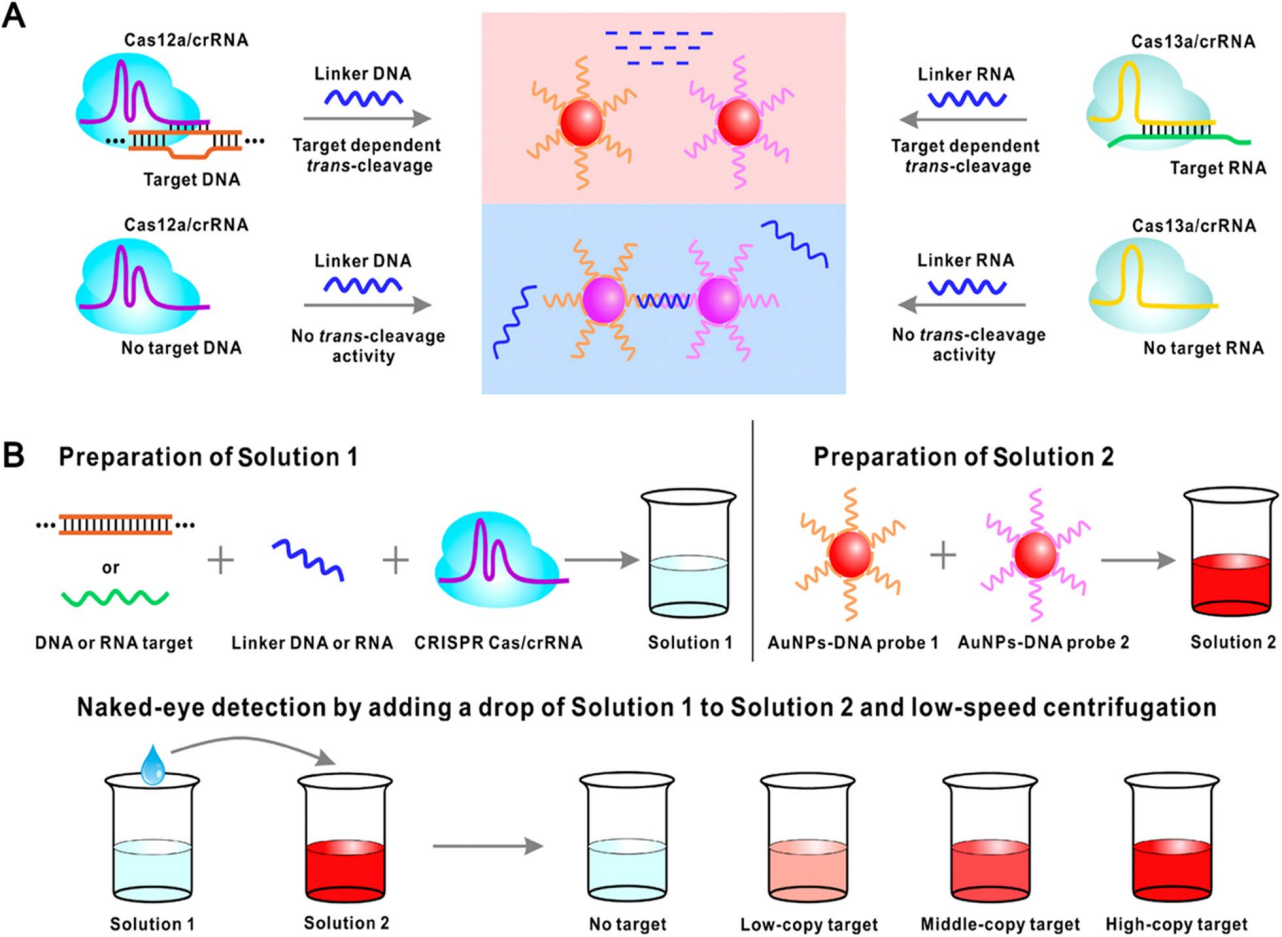
Schematic illustration and workflow of nanoparticle-based CRISPR-Cas system colorimetric gene detection. **A** Signal reporting is based on distance-dependent optical properties of the AuNP–DNA probe pair. In the presence of a target, linker ssDNA or ssRNA is degraded. The AuNP–DNA probe pair loses the hybridization linkers and becomes dispersed. In the absence of a target, linker ssDNA and linker ssRNA remain intact. Cross-linking reaction of the AuNP–DNA probe pair with linker ssDNA or ssRNA results in aggregation. **B** Workflow of CRISPR-based colorimetric gene detection. First, target DNA and RNA are added to Cas/crRNA complexes in the presence of linker ssDNA or ssRNA to prepare Solution 1. The AuNPs–DNA probe pair is mixed to prepare Solution 2. Subsequently, naked-eye detection can be completed by adding a drop of Solution 1 to Solution 2. (Reprinted with permission from [144])

In addition, Ma et al. [141] developed a CRISPR-Cas12a-powered dual-mode biosensor for ultrasensitive detection and cross-validation of pathogens. The system takes advantage of the high sensitivity and specificity of Cas12a and combines colorimetric and photothermal readings using AuNPs. The detection limit was 1 CFU/mL, and the detection range was 10^0^–10^8^ CFU/mL. Using *Salmonella* as a model, they observed the colour changes caused by AuNP probes after CRISPR-Cas12a accurately identified the *invA* gene specific to *Salmonella* and degraded its single-stranded DNAs. Moreover, AuNP-based photothermal detection is less affected by colour in quantitative analysis at 808 nm near-infrared radiation; thus, quantitative measurement can be achieved by using a thermal camera to record the temperature [141]. Yuan et al. [144] designed a colorimetric detection platform combining AuNPs with CRISPR-Cas13a. Based on the 16S rRNA target, the platform can recognize several bacterial pathogens, including *L. monocytogenes*, *S. aureus*, *N. encephalitis*, *Salmonella*, *E. sakazakii*, *P. aeruginosa*, and *V. parahemolyticus*. The system utilizes the optical properties of AuNP aggregation and dispersion, which display colour changes in colloidal solutions to facilitate easy recognition by the naked eye. In addition, the CRISPR-Cas13a system combined with recombinase polymerase amplification (RPA) enables colorimetric measurements to be completed in less than 1 h (Fig. 9) [144].

Lateral flow immunochromatographic assays (LFAs) are paper-based point-of-care (POC) diagnostic tools that are low-cost and convenient. When combined with CRISPR-Cas or other highly specific molecules, they can even achieve high sensitivity and high specificity for POC diagnostics [188]. AuNPs are commonly used as a detection reagent for LFAs due to their stable chemical properties and enhanced visual effect due to their red colour [140, 189]. Mukama et al. [190] developed a DNA probe based on a lateral flow biosensor (LFB) with CRISPR-Cas and loop-mediated isothermal amplification. With the help of the AuNP-SA-biotin-ssDNA reporter complex, a visually visible and portable strip reader was formed. The single copy-level sensitivity of the probe has been demonstrated by the accurate detection of the *P. aeruginosa* acetyltransferase gene and sensitivity to 1 CFU/mL plasmid, and the ability of this method to obtain visible results in less than an hour makes it suitable for on-site tests [190]. CRISPR-Cas12a is also used as a new tool for detecting *H. pylori* in faecal samples, known as CRISPR-HP [191]. Scientists combined the AuNP probe-based LFB method with CRISPR-HP to achieve a simple, convenient result reading that does not rely on complex instruments, which can be applied in epidemiological and large-scale screening studies. The results of CRISPR-HP for *H. pylori* are consistent with qPCR, which shows the advantages of the system in clinical trials. Cas12a-UPTLFA [192] is a highly sensitive and specific portable pathogen diagnosis platform based on the combination of CRISPR-Cas12a and up-converting phosphor technology (UPT)-based gold-based LFA. UPT-LFA based on upconverting phosphor nanoparticles successfully detected *Y. pestis* genomes as low as 3 aM, and the detection of the *pla* gene showed an equivalent detection limit to qPCR, at a minimum concentration of 100 CFU. The fast and convenient testing system is easy to operate, even for nonprofessionals. Wang et al. [193] developed the CRISPR-Cas9-mediated lateral flow acid assay (CASLFA) by combining the excellent dsDNA recognition ability of CRISPR-Cas9 AuNP-DNA probes. CASLFA is a fast, accurate, and portable nucleic acid testing platform with great potential for use in pathogen diagnosis as a point of care test (POCT) in resource-poor or nonlaboratory settings. Scientists used CASLFA to detect *L. monocytogenes* by targeting the *hlyA* gene and demonstrated that the platform could achieve detection limits similar to those of PCR and correctly identify *L. monocytogenes* among five other food-borne pathogens [193].

In addition to AuNPs, Bogers et al. [194] designed Cas12a Activated Nuclease poly-T Reporter Illuminating Particles (CANTRIP) for DNA detection assays. The system combines CRISPR-Cas12a with CuNPs, which have a fluorescence spectrum suitable for detection in complex biological environments (with an emission peak at 625 nm when excited at 340 nm). The presence of target DNA is indicated by the presence of bright orange signals from the CuNPs visible to the naked eye under ultraviolet light. Researchers have demonstrated the potential of CANTRIP by targeting anthrax lethal factor plasmid DNA [194].

## Conclusions and future perspectives

The CRISPR-Cas system has become the most powerful tool ever discovered for gene editing. We believe that the development of novel CRISPR technologies will enable unprecedented control over eliminating drug-resistant bacteria without targeting beneficial bacteria. However, the application of these technologies remains limited by the need for efficient methods for reducing any off-target effects in targeted cells. Selecting an effective delivery system for delivering CRISPR will make it more suitable for clinical interventions, and progress in nanoparticles may provide a better solution. However, nanotechnology-based gene delivery is still in an early stage. Well-designed bioinformatics tools can be used to predict potential off-target binding sites in the genome to improve the design of sgRNA and the structure of Cas enzymes. Tissue-specific promoters and tissue-specific vector delivery systems can also be used to overcome these limitations. In addition, CRISPR-based detection methods have proven to be rapid and highly reliable alternatives to currently used approaches, especially when combined with nanoparticles, and results have indicated great potential for use in the early diagnosis of bacterial infection and resistance. Most CRISPR-based detection techniques require the combination of amplification strategies for target nucleic acids with a known target DNA sequence, which might limit its potential for use in the early diagnosis of infectious diseases caused by unknown pathogens. There is a need for further research efforts that focus on solving these challenges and unlocking the full potential of nanoparticle-based CRISPR technologies to combat and prevent antimicrobial resistance.

## Data Availability

Not applicable.

## Abbreviations

*A. baumannii*: *Acinetobacter baumannii*
ABD: Antibacterial community
AMR: Antimicrobial resistance
ARGs: Antibiotics resistance genes
AuNCs: Gold nanoclusters
*B. cereus*: *Bacillus cereus*
*B. thuringiensis*: *Bacillus thuringiensis*
bPEI: Branched polyethyleneimine
*C. difficile*: *Clostridium difficile*
*C. jejuni*: *Campylobacter jejuni*
CDI: Clostridium difficile Infection
CANTRIP: Cas12a activated nuclease poly-T reporter illuminating particles
CASLFA: CRISPR-Cas9-mediated lateral flow acid assay
CFD: Computational fluid dynamics
CMP: Cas14a1-mediated nucleic acid detection platform
CRE: Carbapenem-resistant Enterobacteriaceae
CRISPR: Clustered regularly interspaced short palindromic repeats
Cr-Nanocomplexes: CRISPR complexes
crRNA: CRISPR-derived RNA
dsDNA: Double-stranded DNA
*E. coli*: *Escherichia coli*
*E. faecalis*: *Enterococcus faecalis*
*E. faecium*: *Enterococcus faecium*
*E. sakazakii*: *Enterobacter sakazakii*
ESBL: Extended-spectrum β-lactamase
*F. novicida*: *Francisella novicida*
gRNAs: Guide RNAs
*H. influenzae*: *Haemophilus influenzae*
*H. pylori*: *Helicobacter pylori*
HGT: Horizontal gene transfer
HUDSON: Heating Unextracted Diagnostic Samples to Obliterate Nucleases
*K. pneumoniae*: *Klebsiella pneumoniae*
*L. monocytogenes*: *Listeria monocytogenes*
LFA: Lateral flow immunochromatographic assay
LFB: Lateral flow biosensor
Lst: Lysostaphin
*M. tuberculosis*: *Mycobacterium tuberculosis*
M-CDC: Magnetic pull-down-assisted colorimetric diagnosis based on the CRISPR-Cas12a system
MDR: Multidrug resistance
MGE: Mobile genetic elements
*N. encephalitis*: *Neisseria encephalitis*
NEase: Nicking endonuclease
*P. aeruginosa*: *Pseudomonas aeruginosa*
*P. gingivalis*: *Porphyromonas gingivalis*
PAM: Protospacer adjacent motif
PCR: Polymerase chain reaction
PFS: Protospacer flaking site
POC: Point-of-care
POCT: Point-of-care test
PRPs: Pheromone-responsive plasmids
qPCR: Quantitative real-time PCR
RPA: Recombinase polymerase amplification
tracrRNA: Trans-activating RNA
TAPs: Targeted antibacterial plasmids
TSB: Tryptic soy broth
TSPE: Target-specific primer extension
UPT: Up-converting phosphor technology
*V. parahaemolyticus*: *Vibrio parahaemolyticus*
WTAs: Wall teichoic acids
*Y. pestis*: *Yersinia pestis*
*S. algae*: *Shewanella algae*
*S. aureus*: *Staphylococcus aureu*
*S. mutans*: *Streptococcus mutans*
*S. pneumoniae*: *Streptococcus* *pneumoniae*
*S. pyogenes*: *Streptococcus pyogenes*
*S.* Typhi: *Salmonella* Typhi
SaPIs: Staphylococcal pathogenicity islands
SERS: Surface-enhanced Raman scatter
sgRNA: Small guide RNA
SNA: Spherical nucleic acid
SNPs: Single nucleotide polymorphisms
SpCas9: Recombinant Cas9 endonuclease from *Streptococcus pyogenes*
STEC: Shiga toxigenic *E. coli*
ssRNA: Single-stranded RNA
